# New Insights into the Exosome-Induced Migration of Uveal Melanoma Cells and the Pre-Metastatic Niche Formation in the Liver

**DOI:** 10.3390/cancers16172977

**Published:** 2024-08-27

**Authors:** Raquel Ramos, Antònia Vinyals, Rafael Campos-Martin, Eduard Cabré, Joan Josep Bech, Javier Vaquero, Ester Gonzalez-Sanchez, Esther Bertran, Josep Ramon Ferreres, Daniel Lorenzo, Carolina G. De La Torre, Isabel Fabregat, Jose Maria Caminal, Àngels Fabra

**Affiliations:** 1 TGF-β and Cancer Group, Oncobell Program, Bellvitge Biomedical Research Institute (IDIBELL), L’Hospitalet de Llobregat, 08907 Barcelona, Spain; ramosmartinraquel@gmail.com (R.R.); avinyals@idibell.cat (A.V.); javier.vaquero@usal.es (J.V.); e.gonzalezsan@usal.es (E.G.-S.); ebertran@idibell.cat (E.B.); joseramonferreresriera@yahoo.es (J.R.F.); ifabregat@idibell.cat (I.F.); 2 Centro de Investigaciones Biomédicas en Red de Enfermedades Hepáticas y Digestivas (CIBEREHD), ISCIII, 28029 Madrid, Spain; 3 Division of Neurogenetics and Molecular Psychiatry, Department of Psychiatry and Psychotherapy, University of Cologne, 50937 Cologne, Germany; rafael.campos-martin@uk-koeln.de; 4 Clinical Proteomics Unit, IDIBELL, 08908 Barcelona, Spain; joanjosepbech@gmail.com (J.J.B.); carolinadelatorre@gmail.com (C.G.D.L.T.); 5 Proteomic Unit, Josep Carreras Leukaemia Research Institute (IJC), Ctra de Can Ruti, 08916 Badalona, Spain; 6 HepatoBiliary Tumors Lab, Centro de Investigación del Cancer and Instituto de Biologia Molecular y Celular del Cancer, CSIC-Universidad de Salamanca, 37007 Salamanca, Spain; 7 Department of Physiological Sciences, Faculty of Pharmacy, University of Salamanca, 37008 Salamanca, Spain; 8 Dermatology Service, IDIBELL, Hospital Universitari de Bellvitge, 08907 Barcelona, Spain; 9 Ocular Translational Eye Research Unit, Ophthalmology Department, Spanish Ocular Oncology National Referral Center (CSUR), Hospital Universitari de Bellvitge, 08907 Barcelona, Spain; dlorenzo@bellvitgehospital.cat (D.L.); jmcaminal@gmail.com (J.M.C.)

**Keywords:** uveal melanoma, exosomes, hepatic stellate cells, macrophages, liver metastasis, mesenchymal migration, amoeboid migration

## Abstract

**Simple Summary:**

Uveal melanoma (UM) is the most common primary intraocular tumor in adults, with a high incidence of liver metastasis causing poor survival. However, the mechanisms underlying this liver tropism remain poorly understood. Our study focused on the roles of UM exosomes along the metastatic process. A comparison of protein cargos between exosomes derived from the primary UM tumor and metastasis reveals a prominent function of exosomes derived from parental cells, favoring the detachment of cells from the primary site. We also found that UM-derived exosomes played a significant role in the activation and transdifferentiation of human hepatic stellate cells (HHSCs), which contribute to the formation of a pre-metastatic niche in the liver, promote interaction with tumor cells, and ultimately might enhance the metastatic potential.

**Abstract:**

UM is an aggressive intraocular tumor characterized by high plasticity and a propensity to metastasize in the liver. However, the underlying mechanisms governing liver tropism remain poorly understood. Given the emerging significance of exosomes, we sought to investigate the contribution of UM-derived exosomes to specific steps of the metastatic process. Firstly, we isolated exosomes from UM cells sharing a common genetic background and different metastatic properties. A comparison of protein cargo reveals an overrepresentation of proteins related to cytoskeleton remodeling and actin filament-based movement in exosomes derived from the parental cells that may favor the detachment of cells from the primary site. Secondly, we assessed the role of macrophages in reprogramming the HHSCs by exosomes. The activation of HHSCs triggered a pro-inflammatory and pro-fibrotic environment through cytokine production, upregulation of extracellular matrix molecules, and the activation of signaling pathways. Finally, we found that activated HHSCs promote increased adhesion and migration of UM cells. Our findings shed light on the pivotal role of exosomes in pre-metastatic niche construction in the liver.

## 1. Introduction

Uveal melanoma (UM) is the most common primary intraocular malignancy in adults, with an estimated annual incidence rate between five and eight cases per million people in the United States and Europe [1]. UM is peculiar for its hematogenous dissemination, with the liver being the most frequent site of metastasis, whose development causes the death of patients [2,3]. Even with recent advances in the management of this disease, patients with liver metastasis still have a poor prognosis. Similarly, adoptive T-cell therapy, or immune checkpoint blockade, has limited responses in UM patients [4]. For this reason, it is urgent to elucidate the molecular events that drive UM metastasis before or at the early stages of hepatic colonization. This would open new opportunities to develop effective therapies capable of preventing or eradicating liver metastasis before it reaches an incurable stage.

Great progress has been made over the last decades in deciphering the genetic landscape of metastatic UM [5], but the reasons for this tropism remain elusive [6]. Emerging evidence indicates that reciprocal interactions between tumor cells and the tumor microenvironment determine the ability of metastatic cells to escape from the primary tumor, enter the circulation, and home on specific organs such as the liver, where they must proliferate and survive [7]. The crosstalk between cancer cells and the different microenvironments is, at least in part, mediated by extracellular vesicles (EVs) secreted by either metastatic or non-metastatic tumor cell populations that act favoring tumor cell extravasation, pre-metastatic niche establishment, and metastatic growth into secondary organs [8,9].

Exosomes, a subset of EVs ranging in size from 30–150 nm, act as cell-free messengers of the primary tumor by transporting their cargo—proteins, DNA, messenger RNA, and microRNAs—to surrounding recipient cells or to a distant site [10,11,12].

Accumulating evidence indicates that proteins secreted [13,14,15] or shed in UM-derived vesicles from cell lines and primary cultures [16,17,18,19] play an important role in tumorigenesis and the establishment of the pre-metastatic niche in the liver.

The UM secretome and exosomes derived from UM cells have been reported to have a key role in UM progression and metastasis [13,14,16,19]. Hence, we first analyze whether exosomes derived from Mel 270 and OMM 2.5 cell lines, which share a common genetic background and different metastatic and phenotypic characteristics [20], may carry different cargo proteins. Second, given the emerging significance of tumor-derived exosomes in mediating the interplay with liver-resident cells [21,22,23], we sought to investigate whether UM-derived exosomes could contribute to the reprogramming of human hepatic stellate cells (HHSCs), creating a favorable milieu in the liver for the growth and colonization of metastatic uveal cells.

## 2. Materials and Methods

### 2.1. Cell Lines and Cell Culture Conditions

The human UM cell lines Mel 270 and OMM 2.5 were provided by Dr. M. J. Jager (Leiden University Medical Center, Leiden, The Netherlands). Cell lines MP41 and MP46 were kindly provided by Dr. J.V. Burnier (McGill University, Montreal, QC, Canada). The human HHSC cell line LX2 and the human monocytic cell line THP-1 were provided by Dr J. Vaquero (Bellvitge Institute for Biomedical Research, Barcelona, Spain).

UM cell lines Mel 270, OMM 2.5, MP41, and MP46 were grown attached to plastic (2D cultures) in Dulbecco’s Modified Eagle Medium/Nutrient Mixture F-12 (DMEM/F-12) media supplemented with 5% of inactivated fetal bovine serum (FBS) and 1% penicillin-streptomycin (5000 U·mL^−1^–5000 μg·mL^−1^) (Life Technologies-Thermo Fisher Sci., Waltham, MA, USA).

The LX2 cell line was grown in DMEM containing 10% FBS. The human macrophage-like THP-1 cells were cultured in RPMI medium supplemented with 20% FBS. All cell lines were maintained at 37 °C in a humidified, 5% CO_2_-enriched atmosphere. Cells were routinely tested for mycoplasma using the PCR technique. Once thawed, the cells were grown for no more than 10 passages and discarded.

To use the THP-1 cell line as a model of Kupffer cells, THP-1 cells were treated with phorbol 12-myristate 13-acetate (PMA) 150 ng·mL^−1^ (P8139, Sigma-Aldrich, St. Louis, MO, USA) for 36 h to induce a macrophage-like phenotype.

Transforming growth factor-β (TGFβ) treatments were performed using 5 ng·mL^−1^ of human recombinant TGFβ1 (Peprotech, Cranbury, NJ, USA) and incubated for the indicated periods.

### 2.2. Viral Production and Cell Infections

Lentiviral plasmid pLenti CMV GFP Addgene#17448 was used as a reporter of EGFP expression (Addgene, Watertown, MA, USA). Lentivirus was packaged as described previously [20]. Supernatants containing viral particles were used for viral infections carried out on exponentially growing cultures in the presence of 8 µg·mL^−1^ polybrene. Two consecutive rounds of infection were performed, and cells were selected with 2 µg·mL^−1^ puromycin (Life Technologies) for two weeks.

### 2.3. Extracellular Vesicles (EVs) Isolation

A total of 6.5 × 10^6^ UM cells were seeded in 150 mm TC-treated culture dishes (Sarstedt, Nümbrecht, Germany) and cultured in complete medium for four days at 37 °C in a humidified 5% CO_2_-enriched atmosphere. The conditioned media (CM) was obtained from serum-starved sub-confluent cultures incubated with serum-free medium for four days. Then the CM was collected and centrifuged at 300× *g* for 10 min to remove dead cells and debris, and subsequently centrifuged at 15,000× *g* for 20 min to remove apoptotic cell bodies and large EVs. Next, the supernatants were filtered through a 0.22 µm filter (Millipore, Burlington, MA, USA) to eliminate vesicles larger than 200 nm. The resulting supernatants were centrifuged at 27,000 rpm for 70 min, and the EV-enriched pellets were rinsed with PBS 1× and centrifuged for another 70 min. Pellets were then resuspended in 200–400 µL of PBS 1×, and protein concentrations were determined using the NanoDrop^TM^ UV–Vis at 280 nm absorbance or a bicinchoninic acid (BCA) assay (Pierce Biotechnology, Waltham, MA, USA). EV-enriched pellets were stored at −80 °C for proteomic analysis.

### 2.4. Cryo-Electron Microscopy of EVs

EVs collected by ultracentrifugation were resuspended at 100 µg/mL in PBS and analyzed by electron cryomicroscopy (Cryo-EM) in the CCiT at the Universitat de Barcelona. Vitrified specimens were prepared by placing one drop of the suspension on the holey carbon surface of a glow-discharged Lacey Carbon 300 mesh copper grid (Ted Pella, Redding, CA, USA). The cryo-immobilization was performed in the Vitrobot Mark III (FEI Company, Eindhoven, The Netherlands) by plunge freezing in liquid ethane-N2(l). The vitrified sample was stored in liquid nitrogen (LN_2_) until its observation.

For imaging, plunge-frozen samples were transferred to a Tecnai F20 EM (FEI, Eindhoven, The Netherlands) using a cryo-holder (Gatan, Pleasanton, CA, USA). The samples were examined at 200 kV at a temperature ranging between −179 and −170 °C. Low-dose images (4096 × 4096 pixels) were recorded on a cooled charge-coupled device (CCD) Eagle camera (FEI, Eindhoven, The Netherlands) with the DigitalMicrograph (Gatan Microscopy Suite, software 2.x (Gatan)).

### 2.5. EVs Labeling and Cell Uptake Assay

EVs isolated from UM cultures were labeled with the green lipophilic fluorescent probe PKH67 (2 µM final concentration) according to the manufacturer’s instructions (Sigma-Aldrich). EV-target THP-1 cells (3 × 10^4^ cells) or LX2 cells (1.5 × 10^4^ cells) were plated on single 24-well plates with round coverslips (Ibidi, Gräfelfing, Germany) and were incubated the day after in serum-free medium with EV-PKH67 dye or controls (PBS- PKH67) for 6–16 h. The dynamin-dependent endocytosis-blocking agent Dynasore, 80 µM (Sigma-Aldrich), was used as a negative uptake control. After the indicated incubation times, cells were rinsed in serum-free medium and fixed in 4% paraformaldehyde for 15 min. To visualize the stained cells, the coverslips were incubated with phalloidin (1:500) (Sigma-Aldrich) for 1 h at room temperature (RT). Next, 4′,6-diamidino-2-phenylindole (DAPI) (1:5000) diluted in PBS 1× was added to stain the nuclei and incubated for 5 min at RT. Finally, stained cells were visualized using a fluorescence microscope (Nikon Eclipse 80i, Tokyo, Japan) at 40× magnification. The images were analyzed by Fiji software (ImageJ, National Institutes of Health, Bethesda, MA, USA, https://imagej.net/ij/ (accessed on 1 April 2024), 1997–2018). The images used in the figures shown are representative of trends observed in all images obtained.

### 2.6. Co-Culture Assay

Briefly, 3.5 × 10^5^ LX2 cells were plated on a single six-well plate (Sarstedt) in complete media and incubated for 18 h. Then, the media was replaced with fresh media containing 0.5% FCS and co-cultured with 7.5 × 10^5^ THP-1 PMA-activated cells in the gelatin pre-coated insert of a Transwell system (0.4 µm pore) (Sarstedt). This pore size allows the diffusion of secreted proteins in the medium but prevents THP-1 cell migration toward the lower chamber. When indicated, 20 µg of exosomes derived from UM cells were added to the upper compartment. The upper compartment of Transwells was filled with serum-free media. After the incubation for the indicated times, the inserts were removed, and the conditioned media was collected and centrifuged at 1200 rpm for 5 min to remove dead cells and debris, and the supernatants were stored at −80 °C. THP-1 and LX2 cell monolayers were collected for RNA and protein analysis and stored at −80 °C until use.

### 2.7. Cell Adhesion Assay

A total of 2.5 × 10^5^ cells from a single cell suspension in serum-free medium of GFP-labeled UM were allowed to attach over a monolayer of untreated LX2 or LX2 cells previously co-cultured with THP-1 in the presence or absence of UM-derived exosomes, as described above. Medium containing 2% FBS was used for attachment to plastic, as a positive control. After incubation for 2 h at 37 °C, cultures were carefully rinsed twice using serum-free medium to remove the nonadherent cells, and wells were filled with fresh serum-free media and incubated for the indicated times. Images were taken using an inverted fluorescence contrast-phase microscope (Olympus IX70) at 10×. Automated counting of GFP-labeled cells was conducted using ImageJ software.

### 2.8. Migration Assays

Firstly, the invasive potential of melanoma spheroids was analyzed using the hanging drop method, as described previously. Briefly, spheroids were resuspended in collagen I (5005; PureCol, Advanced BioMatrix, San Diego, CA, USA) at 1.7 mg·mL^−1^ in DMEM, and after polymerization, cells were incubated in media containing 10% FCS. Phase-contrast images were taken on the fourth day after seeding.

For 3D imaging, fibrillar bovine collagen I was prepared at 1.7 mg·mL^−1^. After polymerization (4 h–37 °C, 10% CO_2_), cells were seeded on top in media containing 10% FBS and fixed after 24 h in culture. Where indicated, cells were starved for 24 h and incubated with 10 µM Blebbistatin (a myosin II ATPase inhibitor) from Calbiochem (Darmstadt, Germany) for the last 2 h. Immunostaining was performed using the antibodies summarized in Supplementary Table S1. For imaging, collagen gels were transferred to glass-bottomed dishes and visualized on a Zeiss LSM 510 Meta confocal microscope (Carl Zeiss, Cambridge, UK) with a C-Apochromat Å~ 40/1.2 numerical aperture (water) and ZEISS ZEN Microscopy Software (RRID:SCR_013672, https://www.zeiss.com/microscopy/en/products/software/zeiss-zen.html, accessed on 1 April 2024) (Carl Zeiss). Confocal Z-slice images were analyzed using ImageJ software.

Cell migration was also assessed in a Transwell system with polycarbonate filters (8 µm pores) (Sarstedt) pre-coated with gelatin. Briefly, cells were starved in serum-free media for 48 h at 37 °C. Where indicated, cells were treated with 20 µg·mL^−1^ of isolated exosomes for 6 h at 37 °C with 40 µM NSC23766 (a Rac1 inhibitor) from Calbiochem. A total of 1.4 × 10^5^ cells were seeded in serum-free media in the upper chamber, and the 600 µL of conditioned media from untreated, exosome-activated LX2 cells or media containing 10% FBS were used as chemoattractants. Conditioned media from LX2 cells treated with TGF-β1 was used in parallel as a positive control. The cells were allowed to migrate through the membrane pores for the indicated times at 37 °C, and those that had not passed through the filter were removed with cotton swabs. The lower surface of the filter was stained with crystal violet solution (0.1% in methanol) for 10 min to detect the cells that had migrated into the lower chamber. After removing excess crystal violet with water, filters were allowed to dry at RT. Cell migration was evaluated using the average number of migrated cells from four random fields (10× magnification). Where indicated, the crystal violet of the stained filters was eluted with 10% SDS, and the absorbance was measured at 570 nm (A^570nm^). Results were presented as the fold change relative to controls.

### 2.9. Proteomic Analysis

#### 2.9.1. Protein Extraction and Digestion of EVs

The isolated exosomes were re-suspended in lysis buffer (8 M urea, Tris 0.1 M supplemented with 1% of complete protease inhibitor cocktail tablets (Roche Applied Sciences, Indianapolis, IN, USA)). To obtain a complete protein extraction, the exosome samples were sonicated in two sonication–cooling cycles (30 s each) and centrifuged at 16,000× *g* at 4 °C for 30 min. After ultrasonic cracking and centrifugation, the protein concentration was measured using the RC DC™ Protein Assay (Bio-Rad, Hercules, CA, USA) kit. Briefly, 10 μg of each sample was digested with Lys-C and trypsin. Before digestion, samples were reduced and alkylated with DTT and CAA, then the samples were diluted with Tris 0.1 M to a urea concentration of 2 M. Lys-C was added at 1:100 (*w*/*w*) (enzyme-to-protein ratio), and protein digestion was carried out at 30 °C. Then the samples were diluted again with Tris 0.1 M to a urea concentration of 0.8 M. Trypsin was added at 1:100 (*w*/*w*) (enzyme-to-protein ratio), and protein digestion was carried out at 30 °C for 8 h. The enzymatic reaction was stopped with formaldehyde (10% (*v*/*v*) final concentration). Peptide mixtures were desalted using the commercial columns (Ultra Microspin C18, 300 A silica, The Nest Group, MA, USA) according to the manufacturer’s instructions. Finally, the samples were dried in a SpeedVac and kept at −20 °C until the LC-MS/MS analysis.

#### 2.9.2. LC-MS/MS Analysis of EVs

One microgram of each sample was analyzed by LC-MS/MS using a 140 min gradient in the Orbitrap Velos Pro (Thermo Fisher Scientific). As a quality control, BSA samples were run between each sample to avoid carryover and to assess instrument performance.

The mass spectrometer was operated in DDA mode, and full MS scans with 1 microscan at a resolution of 120,000 were used over a mass range of *m*/*z* 350–2000 with detection in the Orbitrap. Auto gain control (AGC) was set to 2 × 10^5^ and dynamic exclusion to 60 s. In each cycle of DDA analysis, following each survey scan. Top-speed ions with charges 2 to 7 above a threshold ion count of 1 × 10^4^ were selected for fragmentation at a normalized collision energy of 28%. Fragment ion spectra produced via high-energy collision dissociation (HCD) were acquired in the ion trap; AGC was set to 3 × 10^4^; an isolation window of 1.6 *m*/*z*; and a maximum injection time of 40 ms were used. All data were acquired with Xcalibur software v3.0.63.

#### 2.9.3. Western Blot Analysis

Lysates of EVs and total cell extracts were obtained by adding RIPA buffer containing phosphatase and protease inhibitor cocktail tablets (PhosSTOP and cOmplete Mini EDTA-free) (Roche, Basel, Switzerland). Lysates were centrifuged at 14,000× *g* for 15 min at 4 °C to remove cellular debris. Protein concentration was measured with a BCA assay from Pierce, and 10 µg of protein from EVs or 30 μg of whole cell extract were separated on 10% SDS-polyacrylamide gels. Following electrophoresis, samples were transferred to an Immun-Blot PVDF membrane for protein blotting (Bio-Rad Laboratories, Hercules, CA, USA). Membranes were blocked with 5% (*w*/*v*) nonfat dried milk for 1 h and probed with primary antibodies overnight at 4 °C. The list of antibodies used is provided in Supplemental Table S1.

After washing, membranes were incubated with horseradish peroxidase-conjugated secondary antibodies for 1 h at room temperature. Peroxidase activity was detected with the Immobilon Western Chemiluminescent HRP Substrate (Millipore) following the manufacturer’s instructions. Immunoreactive bands were visualized using an enhanced chemiluminescent system (ChemiDoc, Bio-Rad). Western blot quantifications were performed by densitometry analysis with Image Lab Software version 6.0 (Bio-Rad, RRID:SCR_014210), and relative protein abundance was determined by normalization with β-actin or α-tubulin.

#### 2.9.4. Immunofluorescence Analysis

For immunofluorescence analysis, cells were cultured on 24-well plate coverslips previously coated with gelatin 0.1% (*w*/*v*), fibronectin (5 μg·mL^−1^) or vitronectin (5 μg·mL^−1^) (Invitrogen) and allowed to grow in complete media for 24 h. When indicated, cells were co-cultured with activated THP-1 cells and treated with Mel 270-derived exosomes as described above. After 36 h, coverslips were rinsed twice in PBS 1×, fixed in 4% paraformaldehyde for 15 min at RT, and permeabilized with 0.2% Triton X-100 for 5 min. Coverslips were incubated overnight at 4 °C with the indicated primary antibody. After rinsing twice in PBS 1×, coverslips were incubated for 1 h at RT with the secondary antibody Alexa Fluor 488 (1:1000) (Thermo Fisher Scientific) and phalloidin (1:500) (Sigma-Aldrich) for F-actin staining. Nuclear staining was performed using 4′,6-diamidino-2-phenylindole dihydrochloride (DAPI) (1:5000) (Sigma-Aldrich). Images (20× magnification) were taken using a fluorescence microscope (Nikon Eclipse 80i). For confocal images, collagen gels were transferred to glass-bottom dishes and visualized on a Zeiss LSM 510 Meta confocal microscope (Carl Zeiss, Cambridge, UK) with a C-Apochromat °A 40/1.2 numerical aperture and ZEISS ZEN Microscopy Software (Carl Zeiss). Confocal Z-slice images were analyzed using ImageJ Software (U.S. National Institutes of Health, Bethesda, MA, USA).

#### 2.9.5. Rac1 and RhoA Activity Assay

Cells at 70% confluency were serum starved in serum-free media for 48 h. Cell lysis was carried out with the lysis buffer provided by the manufacturer (Cytoskeleton, Denver, CO, USA). We used PAK-PBD beads to precipitate GTP-bound Rac1 (BK-035) and Rhotekin-RBD to precipitate GTP-bound RhoA (BK-036) from the cell lysate. When indicated, the RhoA activator calpeptin (1 U·mL^−1^) was added to cultures for the last 2 h before lysis. The assays were performed following the instructions provided by the manufacturer (Cytoskeleton). Active and total Rac1 and Rho were separated by 12% SDS-PAGE and visualized by Western blotting using specific antibodies against Rac1 or RhoA. Recombinant His-Rac or His-RhoA protein was used as a positive control in the Western blots.

### 2.10. Total RNA Isolation, Reverse Transcription, and Quantitative RT-qPCR Analysis

Total RNA was extracted from cell cultures or EV pellets using TRIzol reagent according to the manufacturer’s instructions. Reverse transcription was performed with the First Strand cDNA Synthesis Kit (Life Technologies, Carlsbad, CA, USA) using random hexamer primers. Quantitative RT-PCR was performed in an LC 480 machine using the Power UP SYBR Green Master Mix from Roche Life Science (Mannheim, Germany). Gene-specific primers (listed in Supplemental Table S2) were purchased from Life Technologies. Relative gene expression was normalized by the ribosomal protein L32 (RPL32) expression and calculated using the 2^−(*dd*Ct)^ ±SD formula. Assays were performed in triplicate, and results were presented as the fold change relative to controls.

### 2.11. Data Analysis and Biological Functional Network of the EV Protein Cargo

The RAW files were searched with MaxQuant Software (1.6.2.6) using the built-in search engine Andromeda. The samples were searched against a human SwissProt database downloaded in March 2018. Trypsin was chosen as the enzyme, and a maximum of three miscleavages were allowed. Carbamidomethylation (C) was set as a fixed modification, whereas oxidation (M) and acetylation (N-terminal) were used as variable modifications. Searches were performed using a peptide tolerance of 7 ppm and a product ion tolerance of 0.5 Da. The quantification was performed using the LFQ algorithm, and the searches were filtered at a 1% false discovery change (FDR) both at the peptide and protein level. For the rest of the MaxQuant parameters, we used the default values.

The statistical analysis was performed using the statistical software Perseus (1.6.2.1). Briefly, the “proteinGroups.txt” file was filtered to remove the “potential contaminants”, “reverse”, and “identified only by site” entries. Then, the LFQ intensity values were log2 transformed, and the data matrix was filtered to keep only the proteins with valid intensity values in all the samples. A total of 895 proteins were obtained, which were analyzed, and we compared the proteomic cargo of exosomes (EVs) derived from Mel 270 and OMM 2.5. Then we performed a pairwise comparison using a two-sided unpaired Student’s *t*-test and Benjamin statistical analysis. The *p*-values were adjusted using a permutations-based approach, and the significance threshold was set to an adjusted *p*-value < 0.05. Data from each exosome isolation for the two cell lines are presented in triplicate to show sample quality and the degree of experimental reproducibility.

The significant proteins were hierarchically clustered, plotted in a volcano plot, and a hierarchically clustered heatmap in R using the “ggplots2” (https://ggplot2.tidyverse.org) and “Complex Heatmaps” packages version 2.20.0 (https://github.com/jokergoo/ComplexHeatmap, accessed on 1 April 2024).

To delve deep into the biological function of EV proteins, we used Gene Ontology (GO) enrichment analysis to assign each protein to every established GO term, focusing on the Biological Processes (BP) and Cellular Component (CC) categories. In addition to the GO terms, we also performed the functional enrichment analysis using the REACTOME (https://reactome.org/), BIOCARTA, and KEGG (https://www.genome.jp/kegg/, Release 110.0, accessed on 1 April 2024) databases.

A network was generated using the Cytoscape core application version 3.9 (www.cytoscape.org). Differentially expressed proteins in EVs derived from the isogenic cell lines Mel 270 and OMM 2.5 were analyzed in the STRING database (http:string-db.org) to obtain molecular interactions. G-lay clustering with default parameters was performed on the entire network, and then GO-based biological processes were analyzed in each major cluster by Jeppetto in Cytoscape.

We constructed an interaction network based on the STRING database with the differentially expressed proteins from the previous step. Subsequently, analyses were carried out by the Cytoscape core app. Log fold changes in expression between Mel 270G and OMM2.5 cells were used to color map each node/protein. To identify clusters of densely interacting nodes, G-lay fuzzy clustering was applied (clusterMaker2 version 2.3.4. https://apps.cytoscape.org/apps/clustermaker2, accessed on 1 April 2024).

We performed protein set functional association analysis on clusters containing more than 10 proteins using the Jeppetto app. We focused on biological processes and used the STRING database as a background set. Jeppetto computes overrepresentation (ORA) analysis as well as network-based enrichment analysis (EnrichNet) (version 1.1, http://www.enrichnet.org). Especially, EnrichNet computes an XD-score for each ontology, indicating the distance between mapped genes and the pathways in the reference database. Higher values of the score imply a stronger association between genes and pathways.

To determine significant terms, the XD-score was regressed against the corrected q-values of the ORA. Annotations with XD-score values exceeding those estimated for a q-value of 0.05 were selected. Additionally, annotations with q-values higher than 0.5 were excluded. We performed protein set functional association analysis on clusters containing more than 10 proteins using the Jeppetto app. We focused on biological processes and used the STRING database as a background set. Jeppetto computes overrepresentation (ORA) analysis as well as network-based enrichment analysis (EnrichNet). Especially, EnrichNet computes an XD-score for each ontology, indicating the distance between mapped genes and the pathways in the reference database. Higher values of the score imply a stronger association between genes and pathways.

To determine significant terms, the XD-score was regressed against the corrected q-values of the ORA. Annotations with XD-score values exceeding those estimated for a q-value of 0.05 were selected.

Additionally, annotations with q-values higher than 0.5 were excluded.

### 2.12. Statistical Analysis

Data are presented as the mean value ± SD from at least two independent experiments. Statistical analyses were performed with GraphPad Prism version 9.4.0 (453) (GraphPad Software, La Jolla, CA, USA), as well as all histograms and curves. Single comparisons between two groups were determined by the Student’s *t*-test (normal distribution with equal variance). Comparisons between multiple groups were determined by a one-way ANOVA. Statistical significance was considered at *p* < 0.05 (* *p* < 0.05, ** *p* < 0.01, *** *p* < 0.001, **** *p* < 0.0001).

## 3. Results

### 3.1. Isolation and Characterization of UM Exosomes

**Figure 1:**
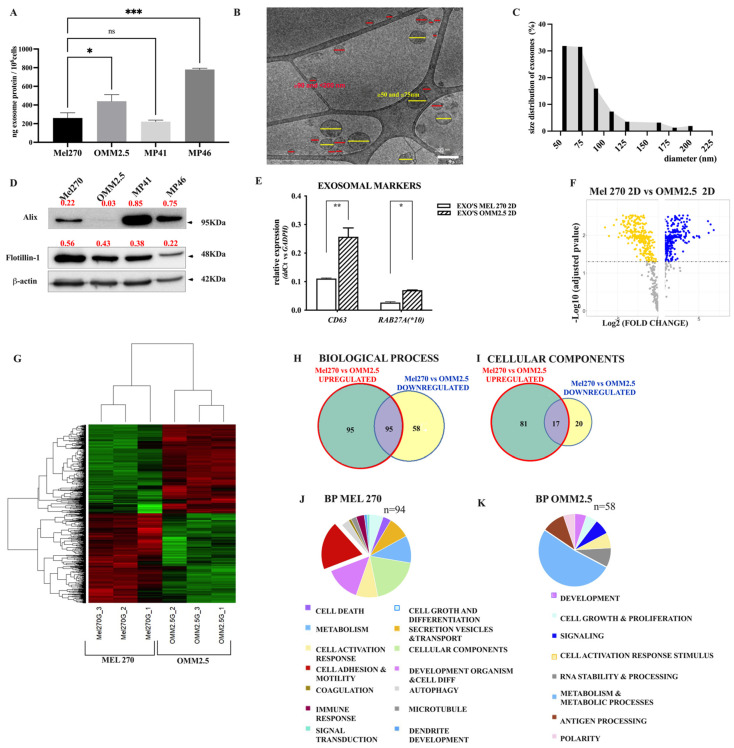
Characterization of EVs secreted by uveal melanoma (UM) cells. (**A**) Production of EVs by different UM cell lines. As described in Material and Methods, EVs were isolated from serum-free cultures of UM cells by ultracentrifugation, and the resulting pellet was quantified for protein content. Bars in the barplot show the amounts of protein (ng) ± SEM of technical replicates in EVs secreted by 10^6^ cells incubated in serum-free media for 60 h. *p*-values were calculated by One-way ANOVA * *p* = 0.05, *** *p* < 0.01, ns (non significant). (**B**) A representative cryo-EM image of EVs isolated from cultured OMM 2.5 cells. Scale bars: 200 nm. (**C**) OMM 2.5- EVs size distribution based on cryo-electron microscopy (cryo-EM) images. Particle measurement conducted using ImageJ software. A total of 500 EVs were analyzed in at least five images. Bars in the barplot show the percentage of EVs segmented by size (nm, in diameter). (**D**) Representative immunoblots for exosomal markers Alix and Flotilin-1 in isolated exosomes isolated from Mel 270, OMM 2.5, MP41, and MP46 UM cells growing in 2D conditions. β-actin was used as a loading control. (**E**) The bars in the barplot show the relative *RNA* expression of *CD63* and *RAB27A* exosomal markers in EVs derived from Mel 270 and OMM 2.5 growing in 2D conditions. mRNA expression was measured by quantitative PCR and normalized by *GADPH*. One representative of three independent experiments is shown and includes the mean ± SEM of technical triplicates. *p*-values were calculated by a two-sided unpaired Student’s *t*-test * *p* < 0.5, ** *p* < 0.05. (**F**) Volcano plot of the log2 fold change Mel 270/OMM 2.5 exosomes (*x*-axis) versus the log 10-corrected *p*-value (*y*-axis), depicting significantly represented exosome proteins. Yellow and blue dots indicate the underexpressed and overexpressed proteins, respectively. (**G**) Heatmap for protein expression in Mel 270 EVs and OMM 2.5 EVs. The Z-transformed expression values for every statistically significant protein (permutations-based adjusted *t*-test *p*-value (FDR) < 0.05) were plotted on the heatmap. Here is a comparison of three independent experimental replicates of Mel 270 and OMM 2.5-derived EVs from 2D cultures. Green colors show the downregulated proteins, red colors the upregulated proteins, and black colors show the median of the expression (log 10 values of the MS signal intensity; scale: <−1 to >1). (**H**,**I**) Venn diagrams indicating the number of overlapping GO terms (purple) of Biological Processes (**H**) and Cellular Components (**I**) overrepresented (up, green) or underrepresented (down, yellow) in Mel 270 and OMM 2.5 exosomes. (**J**,**K**) Pie charts with the percentage of GO terms associated with each GO in Mel 270 and OMM 2.5-derived EVs. (Supplemental Table S5). The percentage indicates the number of proteins assigned to each category relative to the total number of proteins included in the enrichment analysis. BP: Biological Processes. The original Western blot figures can be found in Supplementary File S1.

To characterize the UM exosomes, we isolated EVs from the conditioned media of serum-starved sub-confluent cultures growing in 2D conditions (attached to plastic), using a serial ultracentrifugation method [24]. Exosome yields from the metastatic cell lines OMM 2.5 and MP46 were much greater than those from primary tumor cells (Mel 270 and MP41), as shown by protein content (Figure 1A). Isolated EVs from the OMM 2.5 cell culture supernatant were visualized by cryo-EM (Figure 1B). A total of 500 EVs were analyzed. Those EVs with 60–80 nm diameters (66%) were considered small exosomes, and those of 90–120 nm (26%) corresponded to large exosomes. Bars in the barplot (Figure 1C) show the percentage of EVs segmented by size (nm, in diameter). Similar results were obtained for EVs derived from the other cell lines. We further confirmed these vesicles as exosomes by immunoblot using antibodies against the canonical exosome marker proteins Alix and flotillin-1 (Figure 1D). The presence of Flotillin-1 was detected in all exosomes derived from Mel 270, OMM 2.5, MP46, and MP41 cells, as well as the Alix protein, although the signal observed in OMM 2.5-derived exosomes was very faint. In addition, the mRNA expression of tetraspanin *CD63* and *RAB7A* in EVs derived from Mel 270 and OMM 2.5 cells revealed increased levels of both exosomal markers in OMM 2.5-derived EVs (Figure 1E). Taken together, the results confirmed the identification of the EVs isolated as exosomes.

### 3.2. Differential Proteomic Profiles of Exosomes Derived from UM Metastatic Cells

We analyzed the differential expression of protein cargo in EVs derived from Mel 270 and OMM 2.5 UM cells, which share a common genetic background since OMM 2.5 was derived from a liver metastasis and Mel 270 from the primary tumor of the same patient [25].

EV mass spectrometry data were used for proteomic analyses in a pairwise comparison of Mel 270 vs. OMM 2.5 EVs isolated from 2D cultures in vitro.

We identified 809 proteins containing at least three unique peptides per protein (FDR < 1%). We quantified the expression of proteins by the log2 intensity of signals in three independent EV isolations of each cell type and found 692 significant proteins differently represented between both EV populations (fold change > 2.5) (Supplementary Table S3), and 68 proteins were common in both populations (Supplementary Table S4). Among these, 301 proteins (48.5%) were underexpressed in Mel 270 EVs (yellow dots), while 305 proteins were overexpressed (50.2%) (blue dots) (volcano plot, Figure 1F).

After performing Z-transformation on the significant proteins, hierarchical clustering identified two distinct clusters. In one cluster, proteins were highly abundant in Mel 270 and low in OMM 2.5 EVs. In the second cluster, the pattern was reversed (Figure 1G). To functionally categorize differentially expressed cargo proteins between Mel 270 EVs and OMM 2.5 EVs, we used the DAVID bioinformatics platform. Venn diagrams indicating the number of overlapping GO terms (purple) of Biological Processes (BP) (Figure 1H) and Cellular Component (CC) (Figure 1I); overrepresented (green); underrepresented (yellow) in Mel 270 and OMM 2.5 exosomes.

In Figure 1J,K, the pie charts show the percentage of GO terms of upregulated proteins in Mel 270 EVs and OMM 2.5 EVs belonging to the BP category.

Remarkably, enrichment terms related to cellular organization, differentiation, actin filament organization, and cell–cell adhesion processes were associated with Mel 270 EVs (shown in the red piece of pie), whereas in OMM 2.5 EVs, the most represented terms referred to cell signaling, regulation of metabolic processes, antigen processing, and cell cycle regulation.

Interestingly, the analysis revealed that the abundance of ITGβ_5_, ITGα_3_, ITGβ_1_, ITGα_v,_ and ITGβ_3_ integrins in EVs derived from the metastatic OMM 2.5 cell line was significantly increased compared with that from Mel 270 cells. In addition, the VEGFA:VEGFR2 pathway (R-HSA-4420097) and insulin receptor recycling (R-HSA-77387) were significantly enriched in Mel 270 EVs. Regarding the enriched pathways in OMM 2.5-derived EVs, we highlighted MAPK (R-HSA-5687128), NF*k*B, and TNFR2 (R-HSA-1169091; R-HSA-5668541); Hedgehog (R-HSA-5632684), and cell cycle (R-HSA-69481; GO: 0044770).

**Figure 2:**
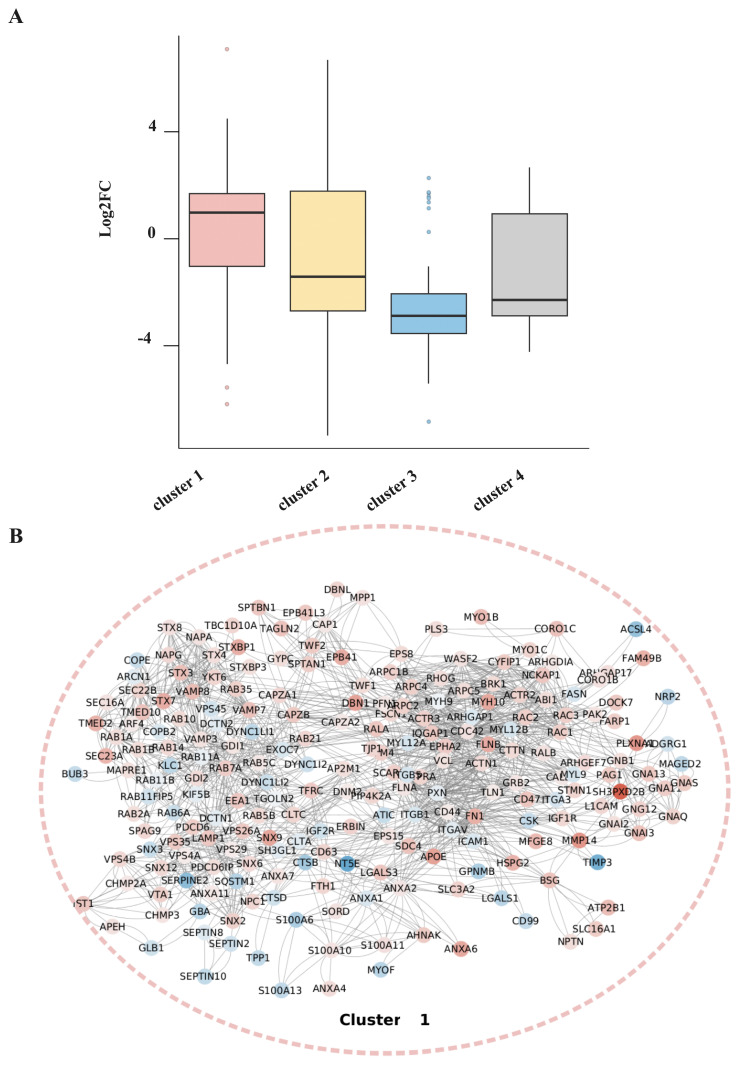

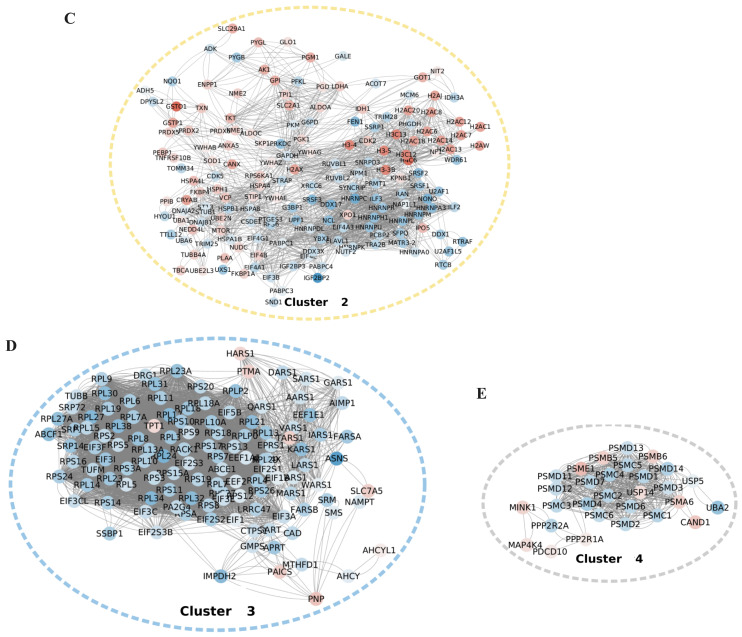
Network analysis showing the interactions between proteins that are significantly overrepresented in Mel 270-derived exosomes compared with OMM 2.5-derived exosomes. (**A**) Four major clusters were identified using G-lay fuzzy clustering. Boxplot of the log2FC values for each cluster. Positive values are proteins upregulated in Mel 270 EVs, and negative values correspond to proteins downregulated. (**B**–**E**) Zoom-in view of the whole protein network for each cluster. Each node corresponds to a protein, color-coded based on log2FC. Red nodes indicate upregulated proteins in Mel 270, while blue nodes are downregulated proteins. Dashed lines delineate Cluster 1 (pink) (panel (**B**)), Cluster 2 (yellow) (panel (**C**)), Cluster 3 (blue) (panel (**D**)), and Cluster 4 (grey) (panel (**E**)).

In parallel, we aimed to identify molecular interaction pathways and biological functions of the EV protein cargo of the Mel 270 and OMM 2.5 EV groups. We performed a network analysis of proteins with significant differences in expression using the STRING database and the Cytoscape application. To gain deeper biological knowledge of the network, we applied G-lay fuzzy clustering to the complete network (631 proteins). This yielded four major clusters with more than 10 proteins (detailed in Supplementary Table S6). The log2FC values for each cluster are shown in the boxplot of Figure 2A, indicating that proteins in Cluster 1 are mostly upregulated in Mel 270 exosomes (mean = 0.783, SD = 1.779), whereas proteins in Cluster 3 are downregulated (mean = −2.237, SD = 1.402).

Network interactions for each cluster are depicted in Figure 2B–E: Cluster 1 (221 proteins, pink), Cluster 2 (169 proteins, yellow), Cluster 3 (120 proteins, blue), and Cluster 4 (29 proteins, grey) (Figure 2B–E). Each protein is color-coded based on the log-fold change in expression: red nodes are overrepresented in Mel 270 exosomes and blue nodes are overrepresented in OMM 2.5 exosomes, with darker colors representing higher absolute values.

The log2FC values for each protein in Cluster1 are plotted in the diagram in Supplementary Figure S1, where the Id is annotated (Ids 1–162 upregulated and Ids 163–221 downregulated proteins).

**Figure 3:**
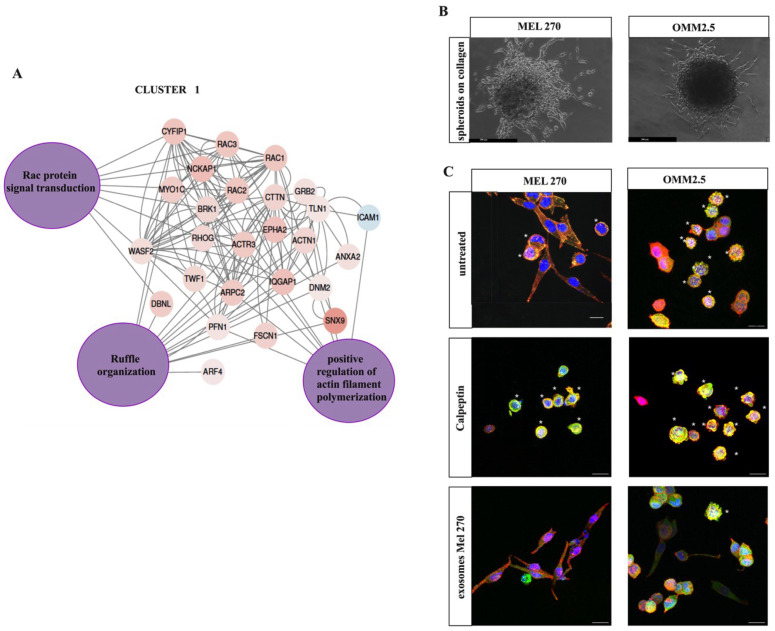

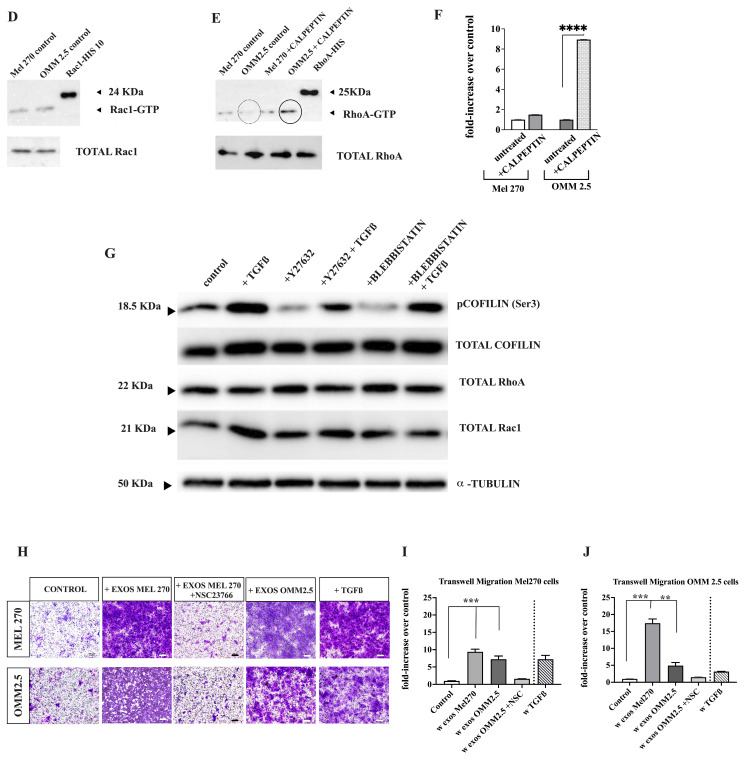
Network analysis showing the interactions between proteins identified in Cluster 1. (**A**) Zoom-in view of the whole protein network for Cluster 1. The identification of this network cluster underscores the significance of cell motility and cytoskeleton organization (see the “purple nodes”). Red labels indicate upregulated proteins in Mel 270, while the blue ones correspond to downregulated proteins. Node names correspond to gene names. (**B**) Invasive growth was analyzed in Mel 270 (**left** panel) and OMM 2.5 cells (**right** panel) by embedding cells as spheroids in the bovine collagen I matrix. The images were taken after seven days of culture and are representatives of two independent experiments. Scale bar: 300 μm. (**C**) 3D collagen invasion assays for Mel 270 (**left** panel) and OMM 2.5 cells (**right** panel). Representative confocal images of F-actin (red), pMLC2 (green), and DAPI (blue). White asterisks indicate cells with blebs. Scale bar: 20 μm. Images from one of four independent experiments are presented. (**D**,**E**) Pull-down assay for Rac1 activity and total Rac1 ((**D**) panel) and RhoA activity and total RhoA ((**D**) panel) in Mel 270 and OMM 2.5 cells on 2D cultures upon starvation for 48 h. Treatments with the RhoA activator Calpeptin (1 U·mL^−1^ for two hours) are indicated. Rac1-GTP and RhoA-GTP were analyzed by pull-down from cell lysates. Positive controls for Rac1 (Rac1-His 10) and RhoA-His were included in the immunoblots. Panel (**F**) shows the quantifications of RhoA-GTP signals from pull-down assay. **** *p* < 0.005. (**G**) Representative immunoblots show that treatments with the TGF-β1 (5 ng·mL^−1^) in OMM 2.5 cells induced an increase in *p*-Cofilin levels without affecting total Cofilin levels, whereas treatment with either ROCK inhibitor Y2732 (10 µM) or Blebbistatin (10 µM) produced a decrease in p-Cofilin levels in both untreated cells and upon the TGF-β1 treatment. Total RhoA and Rac1 levels were unaffected. α-tubulin was used as a loading control. (**H**) Cell migration of Mel 270 and OMM 2.5 cells upon treatment with exosomes (20 µg·mL^−1^) for six hours. When indicated, treatment with NSC 23766 (40 µM) was added. TGF-β1 (5 ng·mL^−1^) treatments were included as a positive control. Results were obtained by a Transwell assay, and values are calculated relative to control (treatment with serum-free media). Panels show the migrated cells on the downside of polycarbonate filters, stained with crystal violet. Scale bar: 100 μm. Images from one of three independent experiments are presented. Quantification of the migrated cells was assessed by measuring the absorbance of the eluted crystal violet. Results are presented as the fold change relative to controls (**I**,**J**). Panel (**I**) shows the migration of Mel 270 cells and panel (**J**) the migration of. OMM2.5 cells. *** *p* < 0.005, ** *p* < 0.05. The original Western blot figures can be found in Supplementary File S1.

The network shown in Figure 3A represents the interactions between proteins significantly enriched in Cluster 1 and their Biological Processes (purple nodes) obtained by Jepetto. The enrichment analysis identified three main nodes related to cytoskeleton remodeling and actin filament-based movement: “Rac protein signal transduction”, “Ruffle organization”, and “Positive regulation of actin filament polymerization”. These features indicate that actin polymerization in Mel 270 cells results in the formation of dorsal ruffles and protrusions at the leading edge of the cell, such as lamellipodia and filopodia, which, together with local matrix proteolysis induced by activated Rac1, mediate the motility of individual cells across the extracellular matrix. This finding corroborated the results above described (Figure 1I) and suggests that Mel 270 cells derived from the primary tumor might preferentially use a “mesenchymal mode of cell migration”. Interestingly, the proteins belonging to these nodes were significantly underrepresented in exosomes derived from the metastatic OMM 2.5 cell line. Other proteins involved in exosome biogenesis, vacuolar protein sorting-associated proteins, transport, and membrane trafficking were also found in Cluster 1, but they are not described here despite their abundance and relevant role in EVs.

Furthermore, Cluster 3 is highly enriched in ribosomal proteins (69/120), and enrichment analysis showed terms such as viral transcription, including proteins involved in liver disease and viral infection by the hepatitis virus, such as PTMA, translational termination, translational elongation, and translational termination (Figure 3D).

Taken together, the network built here allowed the identification of the molecular interaction pathways and biological functions of the EV protein cargo in Mel 270 and OMM 2.5 isogenic cell lines that are relevant for UM progression. Furthermore, the results show that secreted EVs contain distinctive proteins that may contribute differently to distinct steps of the UM metastatic process.

### 3.3. Proteomic Profile and Network of Functional Molecular Interactions of EV Protein Cargo Identify Cell Movement Modes

Previous studies have shown that cell migration and the actin cytoskeleton are key features of UM metastasis [26]. Our proteomic analysis revealed the overrepresentation of biological processes related to cell motility and cytoskeleton (see Cluster 1, Figure 3A), and thus, we further validated the migratory capacity of Mel 270 and the isogenic cell line OMM 2.5.

Firstly, we evaluated the invasive capacity of cells cultured as spheroids embedded in collagen I. This assay measures proliferation and invasion over time (invasive growth) [27].

After seven days of culture, Mel 270 and OMM 2.5 cells exhibited an invasive growth capacity from the center of spheroids, as shown in Figure 3B. Migrated cells showed large lamellipodium protrusions and elongated pseudopodia, classical features of mesenchymal motility driven by Rac1 [28]. Rac1 activates downstream effectors such as WAVE2, which activates the Arp2/3 complex for branches of actin polymerization [29].

We then investigated the cell invasion of UM cells by 3D imaging, where we observed that most Mel 270 cells display spindle-shaped elongation of the body cell, reminiscent of mesenchymal-like morphology, and only 20% of cells showed a round morphology. In contrast, the majority of OMM 2.5 cells exhibited a round morphology with prominent bleb-like structures, suggesting that cells use actomyosin contractility to move under these conditions (Figure 3C). Amoeboid motility has been reported to be driven by RhoA, which activates Rho-associated protein kinases ROCK1 and ROCK2, resulting in increased actomyosin contractility via myosin light chain phosphorylation (pMLC2) localized in blebs [30].

Accordingly, by Rac1 and RhoA pull-down assays, we observed a signal with similar intensity for endogenous Rac1 couplet to GTP as well as RhoA coupled to GTP in both Mel 270 and OMM 2.5 (Figure 3D,E). We found that active RhoA was increased after treatment with the RhoA activator Calpeptin in both cell lines, and particularly in OMM 2.5 cells (Figure 3E, *dashed circle*: untreated; *full circle*: Calpeptin treated). The densitometric analysis of RhoA after Calpeptin treatment is shown in Figure 3F. This finding was further corroborated by confocal images, in which we detected an increased number of blebs (Figure 3C). Moreover, a phenotype switch was detected in 70% of Calpeptin-activated Mel 270 cells, which significantly reduced elongation and adopted a round morphology with some blebs, suggesting the ability of these cells to allow the mesenchymal to ameboid transition.

For instance, total levels of Rac1, RhoA, and Cofilin-1 in OMM 2.5 cells were unaffected by the treatments with either the ROCK inhibitor Y-27632 or Blebbistatin, which presumably affect the active RhoA since the levels of its downstream phosphorylated substrate Cofilin-1 were diminished compared with control cells (Figure 3G). Interestingly, both inhibitors also reduced the levels of TGFβ-induced p-Cofilin-1. Of note, we did not observe such changes in parallel experiments with Mel 270 cells.

Notably, as shown in Supplementary Figure S2, we did not detect any significant differences in the activity of secreted gelatinases MMP-9, MMP-2, and membrane-associated MMP14 (MT1-MMP) in 2D cultures, which could contribute to collagen degradation and individual cell migration. Therefore, and according to the confocal images, this indicates that when required, both cell lines are candidates to migrate in an MMP-dependent manner.

Finally, we tested if exosomes released by the Mel 270 (derived from a primary tumor) or the OMM 2.5 metastatic cells might induce migration in homologous or heterologous UM recipient cells. To this end, cells were starved in serum-free media for 48 h before treatment with exosomes. Cells were incubated with exosomes or serum-free media for 6 h and then, seeded in the upper chamber of Transwells. Our data show that exosomes from both cell lines significantly enhanced the migration of both isogenic variants compared with controls (Figure 3H). Moreover, the exosomes derived from Mel 270 induced a high migration effect on OMM 2.5 cells, suggesting that EV protein cargo from parental cells might favor the OMM 2.5 mesenchymal mode of migration. Accordingly, the Rac1 inhibitor NSC23766, which prevents the interaction of the GTP/GDP exchange factors with Rac1, significantly reduced the number of migrated cells (Figure 3I,J). The shift toward a mesenchymal mode of migration was also observed when we exposed the exosome-activated UM cells to three-dimensional matrices of collagen I. As shown in Figure 3C, some cells adopt an elongated morphology with lamellar and lamellipodial protrusions.

Altogether, our results indicate that both Mel 270 and OMM 2.5 cells can use both mesenchymal and amoeboid modes of migration because of their plasticity. Importantly, Mel 270 parental cells preferentially migrate through Rac1 signaling, whereas the metastatic OMM 2.5 cells migrate through RhoA activation and display an amoeboid phenotype. However, under microenvironment conditions or treatment with Rac1-enriched exosomes, they switched their migratory phenotype [31,32].

### 3.4. Proteomic Analysis of UM-Derived EVs Reveals a Distinct Integrin Profile in Metastatic and Non-Metastatic Cells

**Figure 4:**
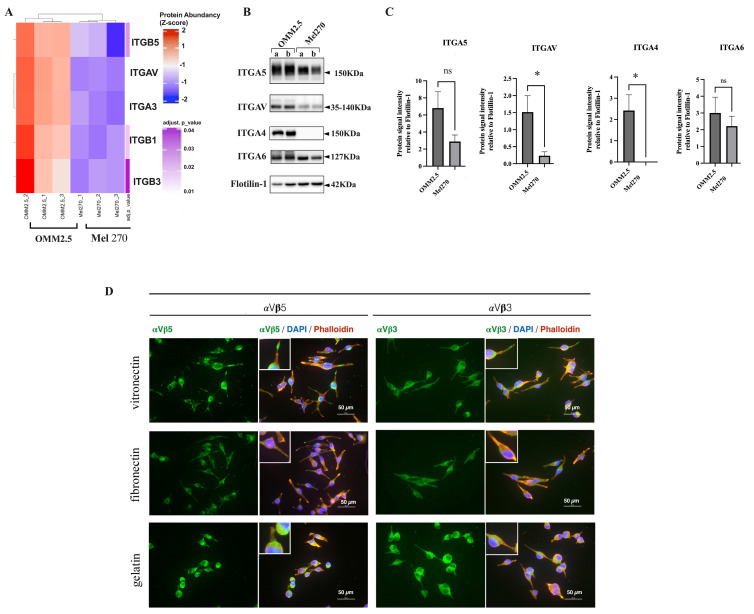
Heatmaps depicting the relative expression levels of proteins belonging to “Integrin signaling pathway”. (**A**) Heatmap shows the relative expression levels of proteins belonging to “Integrin signaling pathway” (P00034) detected in exosomes derived from OMM 2.5 and Mel 270. Here is shown the comparison of three technical replicates. (**B**) Representative immunoblots of ITGα5, ITGαV, ITGα4, and ITGα6 integrins in EVs from OMM 2.5 and Mel 270. Equal amounts (30 µg) of exosome proteins were probed with the indicated antibodies against integrins. Two experimental replicates of EVs were loaded, and the Flotilin-1 signal was used as a loading control. Panel (**C**) shows the quantifications of signals detected in their respective immunoblots. *p*-values were calculated by two-sided *t*-test * *p* < 0.05; ns non-significant. (**D**) Representative images of integrins α_v_β_5_ (green) and α_v_β_3_ (green) immunostaining in OMM 2.5 cells seeded on vitronectin, fibronectin, or gelatin. Slides were counterstained with phalloidin (red) and DAPI (blue). Scale bar: 50 µm. One representative of three independent experiments is shown. The original Western blot figures can be found in Supplementary File S1.

Exosomes need to interact with recipient cells to transfer their contents, and target cell specificity for exosome binding is likely to be determined by adhesion molecules, such as integrins [33]. The heatmap chart in Figure 4A is a detailed version of the heatmap shown in Figure 1G, in which the abundance of ITGβ_5_, ITGα_3_, ITGβ_1_, ITGα_v_, and ITGβ_3_ integrins in EVs derived from the metastatic OMM 2.5 cell line was significantly increased compared with that from EVs derived from Mel 270 cells. We then validated these results by immunoblot analysis, corroborating robust ITGα_V_ and ITGβ5 expression in exosomes derived from the metastatic cell line (Figure 4B). In addition, we reprobed the same membrane with antibodies against ITGα4 and ITGα6, which have been shown to guide circulating tumor cells to the lungs [33], and we also found an increased signal in EVs derived from the metastatic OMM 2.5 cell line compared with the expression in Mel 270-derived EVs. Importantly, in terms of the integrin expression profile, our UM-derived exosomes reflected the biological content of the cells they were derived from.

Furthermore, the immunofluorescence analysis of OMM 2.5 cell cultures showed how α_v_β_5_ and α_v_β_3_ focal adhesions were formed in the presence of the extracellular matrix (ECM) proteins vitronectin (VN) and fibronectin (FN) but not with gelatin (Figure 4D). This further confirms our proteomic results and highlights the specificity of these ECM receptors.

### 3.5. Exosome Uptake by Hepatic Stellate Cells In Vitro

**Figure 5:**
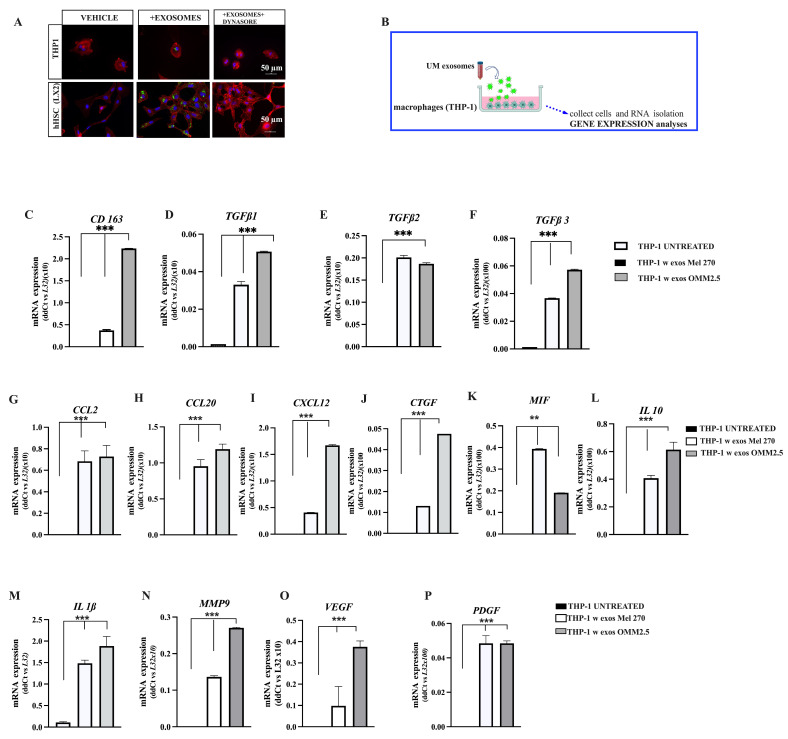
Activation of THP-1 macrophages by exosomes derived from UM cells leads to an increase in transcripts of TGF-β1 isoforms, cytokines, and growth factors involved in liver fibrosis. (**A**) Uptake and internalization of UM exosomes by hepatic cells. Representative immunofluorescence images of THP1 (**upper** panels) and human stellate cells (HHSCs) LX2 cells (**bottom** panels). The (**left** panels) show cells treated only with vehicle (DMSO). PKH67-labeled exosomes (green) from OMM 2.5 cells were incubated with THP-1 or LX2 cells for 16 h and subjected to immunofluorescence. When indicated, cells were treated with the Dynamin-2 inhibitor (Dynasore), (**right** panels). Cell nuclei were stained with DAPI (blue), and slides were counterstained with phalloidin (red). Scale bars: 50 µm. (**B**) Schematic illustration of THP-1 cells in culture. Briefly, 7.5 × 10^5^ PMA-activated THP-1 cells were cultured in 0.5% FBS. When indicated, 20 µg of exosomes derived from UM cells, was added and incubated for 36 h, and then cells were collected for further analysis. (**C**) Novel expression of the M2 marker *CD-163* was detected in the exosome-treated macrophages (THP-1). (**D**–**F**) The production of *TGFβ* isoforms was detected in THP-1 cells treated with uveal exosomes. (**G**–**L**) Treatment of THP-1 macrophages with uveal-derived exosomes triggered a significant increase in chemotactic cytokine transcripts and their receptors, such as *CCL2* (**G**), *CCL-20* (**H**), and *CXCL12* (**I**), as well as the pro-fibrotic *CTGF* (**J**) and the macrophage inhibitor factor *MIF* (**K**). We also observed an induction of the common biomarker for M1 and M2 macrophages, *IL-10* (**L**), upon treatment with exosomes. (**M**–**P**) THP-1-derived macrophages treated with uveal exosomes increased the expression of bioactive molecules associated with angiogenesis and metastasis, such as *IL-1β* (**M**), *MMP-9* (**N**), *VEGFA* (**O**), and *PDGF* (**P**). Data from at least three independent experiments are represented as the mean ± SEM. *p*-values were calculated by one-way ANOVA; ** *p* < 0.05, *** *p* < 0.005.

As the delivery of exosome content requires exosome internalization by target cells, we next evaluated if the human macrophage cell line THP-1 and the HHSCs (LX2 cell line) were able to uptake UM-derived exosomes. For this purpose, exosomes isolated from the metastatic UM cell line OMM 2.5 were labeled with the fluorescent dye PHK67, as described in Material and Methods. After incubation of these labeled exosomes with THP-1 and LX2 cells, we detected fluorescent particles in the perinuclear region or the nucleus of cells, suggesting that exosome internalization might induce transcriptional changes in the recipient cells. Remarkably, treatment of cells with the dynamin inhibitor Dynasore impaired the exosome uptake by THP-1 and LX2 cells (Figure 5A).

In addition, we found that UM-derived exosomes were internalized faster by THP-1 cells, as we could detect the intracellular presence of exosomes in just 6 h, while they were seen in LX2 cells only after 16 h. This may suggest that macrophages are the predilect cell type for the uptake of UM-derived exosomes, contributing directly or indirectly to the pre-metastatic niche formation in the liver.

### 3.6. UM-Derived Exosomes Induce a Pro-Fibrotic and Inflammatory Phenotype in HHSC

Once we verified that UM-derived exosomes can be internalized by THP-1 and LX2 cells, we proceeded to analyze their effects on these cells. To this end, we first explored the effect of isolated EVs by directly adding 20 µg·mL^−1^ of exosomal protein to the culture media of PMA-stimulated THP-1 cells, as shown in Figure 5B. After 36–48 h, THP-1 cells were collected by centrifugation and used for gene expression analysis. A significant upregulation of the non-inflammatory hemoglobin/haptoglobin receptor *CD16*3 and *IL10* transcript levels (Figure 5C,L), both known markers of the M2 phenotype [34], was detected after exposition to Mel 270-, OMM 2.5-, and MP41-derived exosomes. Importantly, we also detected significantly increased levels of *TGFβ-1*, *2*, and 3 transcripts (Figure 5D, Figure 5E and Figure 5F respectively), which might create a favorable pro-tumoral microenvironment.

According to previous reports, the above UM exosomes polarize THP-1 macrophages towards an immunosuppressive M2 phenotype [35,36]. Additionally, macrophages treated with UM exosomes had significantly increased transcript levels of bioactive molecules compared with controls in the absence of exosomes: chemokines (*CCL2*, *CCL20*, and *CXCL12*, Figure 5G–I), *MIF* (Figure 5K)*,* growth factors (*CTGF* and *PDGFB,* Figure 5J,P), cytokines (*IL-10* and *IL-1β*, Figure 5L,M), and *MMP-9*, *VEGFA*, (Figure 5N,O).

**Figure 6:**
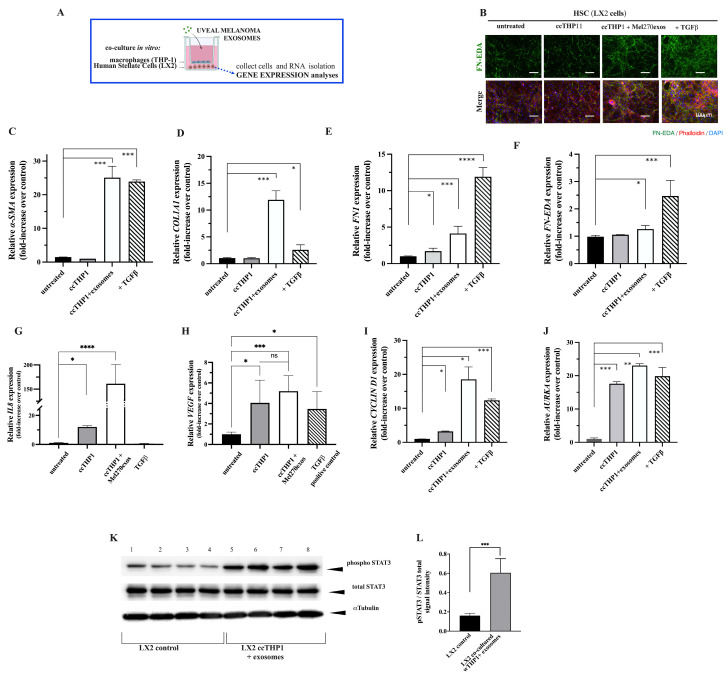
Exosomes derived from UM cells co-cultured with macrophages induce a fibrotic and proangiogenic phenotype in hepatic stellate cells. (**A**) Schematic illustration of co-cultures of HHCSs and THP-1 cells. Briefly, 3.5 × 10^5^ LX2 cells per well were plated on a six-well plate in complete media and incubated for 18 h. Then, the media was replaced with fresh media containing 0.5% FBS and co-cultured with 7.5 × 10^5^ PMA-activated cells (THP-1) in the insert of a Transwell system (0.4 µm pore) for additional 36 h in the presence of uveal exosomes (20 µg/mL). This pore size allows the diffusion of secreted proteins in the medium but prevents THP-1 cell migration toward the lower chamber. After this time, HHSCs were collected, and RNA was obtained for gene expression studies. (**B**) The exosomes derived from Mel 270 cells induce a pro-fibrotic phenotype in HHSCs co-cultured with the activated THP-1 cells. Expression of the FN-EDA in LX2 growing alone or under co-culture conditions in the presence or absence of UM-derived exosomes, as determined by immunofluorescence. LX2 cells treated with TGFβ-1 were included as positive control for FN-EDA. Scale bars: 100 µm. (**C**) mRNA expression of α*-SMA*, *COL1A1* (**D**), *FN1* (**E**), and *FN-EDA FN1* (**F**) was analyzed in HHSCs by quantitative PCR and normalized by *L32*. (**G**) The expression of cytokines *IL-8* and *VEGFA* (**H**) is shown in the indicated boxplots. The expression of cell-cycle markers *Cyclin D1* and *AURKA* is shown in the boxplots (**I**,**J**), respectively. All qPCR results are presented as fold increase relative to the expression of LX2 cells growing alone on plastic. Treatments of LX2 cells with exogenous TGF-β1 were included as positive controls. One representative of three independent experiments is shown in all cases. All bars in barplots show the mean ± SEM of technical triplicates. *p*-values were calculated by one-way ANOVA; * *p* < 0.05, ** *p* < 0.01, *** *p* < 0.001, **** *p* < 0.0001, ns: non-statistical significant. (**K**) A representative immunoblot of phosphorylated and total STAT3 in LX2 cultured alone (lanes 1–4) or in co-culture with THP-1 cells (lanes 5–8). α-tubulin was used as a loading control. Four independent replicas for each condition are shown. The quantification of the pSTAT3/STAT3 ratio is shown in the next panel (**L**). *p*-values were calculated by two-sided *t*-test. The original Western blot figures can be found in Supplementary File S1.

Then, we set up a co-culture system in which the addition of exosomes to THP-1 cells might induce phenotypic changes in LX2 cells. A schematic representation of the Transwell system designed is shown in Figure 6A. Morphological changes in LX2 cells and increased expression of FN-EDA were detected upon exosome treatment (Figure 6B). After incubation for 36–72 h in the presence or absence of UM exosomes, we collected LX2 cells from the lower compartment, and the mRNA abundance was analyzed by RT-qPCR. First, we analyzed the expression of α-smooth muscle actin (*α-SMA*), which has been associated with the transdifferentiation of HHSCs [37]. As shown in Figure 6C, a significant increase was observed in *α-SMA* upon treatment with exosomes as compared with control cells. Similar levels of *α-SMA* expression were observed after the treatment of LX2 cells with exogenous TGF-β1 (2 ng·mL^−1^). Likewise, the expression levels of *COL1A1* were found to be significantly increased in THP-1/LX2 co-cultures in the presence of exosomes (Figure 6D). We then explored the *FN* and *FN-EDA* (FN containing extra domain A) expressions, which are known to stimulate FN production by HHSCs [38,39]. We detected a significant increase in *FN* and *FN-EDA* production by LX2 cells co-cultured with THP-1 cells in the presence of UM exosomes compared with control cells (Figure 6E,F).

Of note, incubation of LX2 cells with exosomes in the absence of co-culture with THP-1 cells or in the presence of TGFBR inhibitor did not induce the pro-fibrotic effect on LX2 cells (Supplementary Figure S3).

It is known that activated HHSCs produce many inflammatory cytokines that have been shown to play key roles in regulating liver fibrogenesis and angiogenesis [40]. Thus, we asked whether LX2 cells activated by UM exosomes may induce the expression of some cytokines. We highlight the induction of the interleukin-8 (*IL8*) transcript after the treatment of co-cultures with exosomes (*p* < 0.0001). Of note, low levels of this transcript were detected in co-cultures but not in control cells or upon TGF-β1 treatment (Figure 6G).

Exosome treatment also increased the mRNA expression of the angiogenic vascular endothelial growth factor (*VEGFA*) compared with the control (*p* < 0.001) (Figure 6H).

Furthermore, we explored the expression of *CYCLIN D1* and *AURKA* by LX2 co-cultures treated with exosomes and found a significant increase in both proliferation markers as compared with LX2 controls, albeit the co-culture with THP-1 per se induced similar *AURKA* levels (Figure 6I,J). Interestingly, elevated *CYCLIN D1* mRNA levels upon exosome treatments correlated with activated STAT3 proteins in LX2 cells, as shown in the Western blot of Figure 6K. The densitometric analysis is shown in Figure 6L.

Collectively, these findings suggest that UM-derived exosomes, with the involvement of macrophages, contribute to the reprogramming of HHSCs, generating a pro-fibrotic and pro-inflammatory microenvironment in the liver that might favor the homing of the metastatic UM cells.

### 3.7. UM Exosome-Treated LX2/THP-1 Simulates Cell Adhesion and Migration of UM Cells

**Figure 7:**
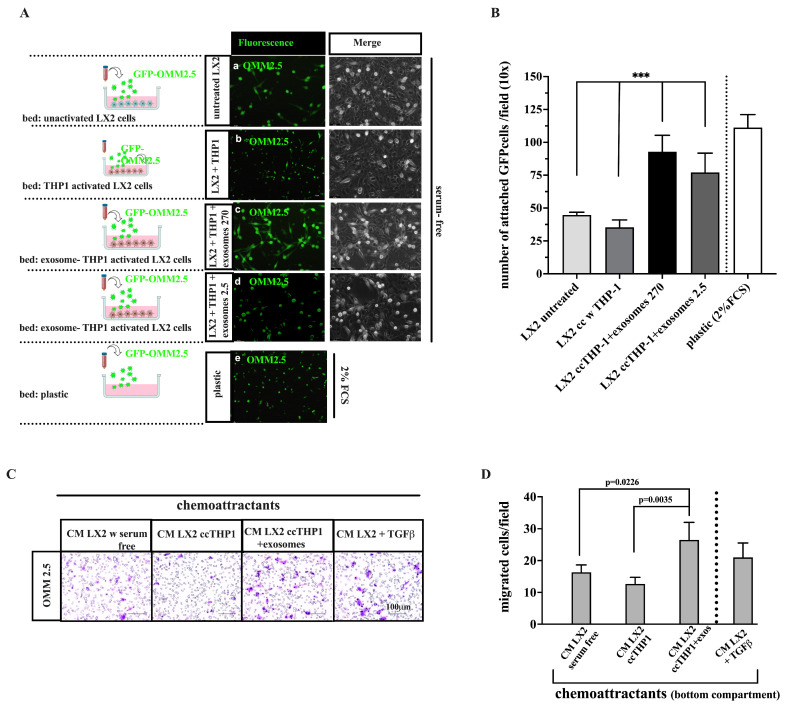
Activated hepatic stellate cells by exosomes derived from UM cells increase the attachment and migration of tumor cell spheroids. (**A**) Enhanced adhesion of UM cells to activated HSC (LX2) by exosomes. The schematic workflow for the adhesion experiments is shown in the (**left** panel). GFP-labeled OMM 2.5 cells were plated either on untreated LX2 or on LX2 activated by co-culture with THP-1 cells in the presence or absence of exosomes (20 µg·mL^−1^). Single-cell suspensions of GFP-labeled UM cells were prepared in serum-free media and plated on confluent HHSC beds. After 2 h, the non-attached cells were removed, and cultures were incubated for an additional 6 h. Direct adhesion of GFP-labeled OMM 2.5 on plastic, but in the presence of 2% FBS, was used as a positive control. The number of attached GFP cells was quantified by ImageJ software from microphotograph images from five independent fields. Representative fluorescence and contrast-phase images of each condition are shown. Scale bars, 50 µm. (**B**) The bars in the boxplot show the percentage of attached cells on each bead. One representative of three independent experiments is shown and includes the mean ± SEM of five independent fields per condition. *p*-values were calculated by one-way ANOVA; *** *p* = 0.0002 compared with the adhesion on untreated LX2 cells. (**C**) Exosome-activated HHSCs (LX2) induce the migration of UM cells. Cell migration of OMM 2.5 cells was assessed by Transwell assay using polycarbonate filters (8 µm pores). Briefly, 1.4 × 10^5^ cells were seeded in serum-free media in the upper chamber, and the conditioned media from untreated or activated LX2 cells was used as a chemoattractant (bottom compartment). Conditioned media from LX2 cells treated with TGF-β1 was used in parallel as a positive control. Panels show representative microphotographs of migrated cells on the downside of polycarbonate filters after 16 h of incubation and staining with crystal violet. Scale bar: 100 µm. (**D**) Quantification of migrated cells. The bars in the barplot show the average number of migrated cells from four random fields (X10 magnification) (mean) ± SEM. *p* was calculated by a two-sided unpaired Student’s *t*-test. One of three experiments is presented.

We investigated whether the attachment of UM cells to HHSCs may be influenced by treatment with UM-derived exosomes. To this end, OMM 2.5 and Mel 270 cells were labeled with EGFP and then plated over the LX2 monolayers co-cultured with THP-1 cells and pre-treated with UM-derived exosomes. The results showed that the number of OMM 2.5 spheroids attached to LX2 cells was significantly increased compared with untreated LX2 cells (*p* = 0.0002, Figure 7A,B).

To assess whether the migratory capacity of UM cells may be influenced by exosome-mediated activation of HHSCs, the conditioned media of LX2 co-cultures in the presence or absence of UM-derived exosomes was used as a chemoattractant in the lower compartment of a Transwell^®^ system. Single suspensions of OMM 2.5 or Mel 270 cells in serum-free media were plated on the upper compartment. After incubation for the indicated times, we observed that the number of cells that migrated toward the conditioned media obtained in the presence of exosomes was significantly higher compared with controls using conditioned media either from LX2 alone or co-cultured with THP-1 cells as chemoattractants (Figure 7C,D). These results indicate that chemokines and cytokines released into the media of co-cultures in the presence of UM exosomes enhanced the migration of UM cells.

## 4. Discussion

### 4.1. Proteomic Analysis of EVs Secreted by UM-Cells Reveals Key Proteins Related to Migration and Invasion

In the present study, we compared the proteomic cargo of EVs derived from UM primary tumor and metastasis cell lines established from the same patient. EVs isolated from UM cells were confirmed by the positive expression of canonical markers and size [41]. As compared with Mel 270, we obtained a higher exosome yield from the OMM 2.5 metastatic cell line and the MP46 carrying a *BAP1* mutation, which is associated with metastasis. This finding suggests that an increased number of exosomes in circulation may promote the development and spread of tumors [42].

When we investigated the differentially represented cargo proteins in EVs secreted by Mel 270 and metastatic OMM 2.5 cells, we found that enrichment terms related to actin organization, cell–cell adhesion processes, cytoskeletal dynamics, and particularly, melanoma biology were among the most abundant components in EVs derived from Mel 270 cells, while the most overrepresented proteins in EVs from OMM 2.5 cells belonged to the MAPK, NF-*k*B, and Hedgehog signaling pathways, which may sustain the cell migration abilities already acquired by the metastatic cells.

The functional interaction network identified interconnected proteins grouped in Cluster 1 involved in migration and invasion. Among them, we particularly focused on those related to cytoskeletal organization and cell locomotion, known as “regulation of actin and filament organization” which were overrepresented in Mel 270-derived EVs (Figure 2A). Actin and actin-associated proteins related to polymerization machinery, myosin mechanoenzymes, and proteins that stabilize or depolymerize microfilaments are included in this cluster [43]. A second node is the “ruffle organization”, in which members participate in the formation of protrusions and other plasma membrane structures. The third node connects the components of “Rac signal transduction”, whose contribution to cell movement is broadly accepted [44]. Remarkably, in Cluster 1, we also found overrepresented the CDC42 protein, which shares common effectors with Rac members and, among other functions, contributes to JKN activation and actin polymerization [45].

During cancer progression, tumor cells acquire the ability to disseminate from the primary tumor, and this process includes ECM remodeling to cross the tissue’s interstitial collagen barriers. To this end, tumor cells secrete ECM-degrading enzymes preferentially at the “invadopodia”, or actin-rich subcellular structures that protrude into and break down ECM components. The metalloproteinase MT-MMP (MMP14), known to enhance the cell migration and invasiveness of UM cells [46], was found to be overrepresented in Mel 270-derived exosomes. Interestingly, Hoshino et al. have shown that invadopodia maturation and ECM degradation are dependent on the delivery of MT1-MMP via exosomes [47]. MT1-MMP and Cdc42, also overrepresented in our exosomes, are part of the invasion–signaling complex that controls directed single-cell invasion of 3D collagen matrices [48]. Furthermore, Zhuge and Xu demonstrated that MT1-MMP, which enables the activation of MMP-2, is transcriptionally regulated by Rac1, and both MMPs enact the Rac1-attributed cell invasion [49]. Rac1 is a member of the Rho family of proteins that regulates the assembly of a meshwork of actin filaments at the cell periphery to produce lamellipodia and invadopodia, related to the mesenchymal mode of migration [50,51]. Rac1 is also overrepresented in EVs derived from Mel 270 cells, suggesting that they may preferentially guide the mesenchymal migration from the primary tumor. Interestingly, we also found an overrepresentation of the RhoG protein, a negative regulator of invadopodia formation [52], and IQGAP1, which contribute to suppressing the local activity of Rac1 [53]. The inactivation of Rac1 promotes RhoA activation and actomyosin contractility, and cells adopt amoeboid features such as blebs and microvesicles [51,54]. The balance between levels of activated Rac and Rho determines the mesenchymal or amoeboid mode of cell motility [55]. Confocal images revealed that both isogenic cell lines may adopt either a mesenchymal or amoeboid phenotype. However, Mel 270 cells preferentially migrate via a mesenchymal mode, whereas the OMM 2.5 cells on the collagen invasion assay appear round, and most of them exhibit blebs at the cell surface where the phosphorylated Myosin light chain (pMLC2) is localized. Moreover, we observed that either the treatment of OMM 2.5 cells with Rac1-enriched exosomes or conditioned media from transactivated HHCSs by exosome-treated macrophages significantly induces the number of migrating UM cells, which adopt a mesenchymal phenotype. In contrast, other studies on cancer exosomes, such as neuroblastoma, demonstrated that amoeboid cell movement is restricted to metastatic cells and mediated by exosomes derived from metastatic cell lines [56]. Furthermore, we did not have evidence that EVs derived from the metastatic cell lines OMM 2.5 or MP-41 induce a phenotypic switch on Mel 270 cells from elongated to rounded amoeboid, as Schillaci and colleagues described in isogenic metastatic and non-metastatic colon carcinoma cells [57]. In agreement with previous reports on cutaneous melanoma [30,31], our observations herein indicate that UM cells adopt features of both mesenchymal and amoeboid migration in a dynamic manner depending on the characteristics of the environment through which they must migrate, a feature called migration plasticity [58,59]. Although the mesenchymal mode is required for cell migration from the primary, the acquisition of adhesion-independent locomotion is advantageous for cells traveling fast through low-adhesion environments such as blood or lymph without ECM degradation and increased survival conveyed by blebbiness [60]. However, when cells need to cross tissue barriers, intercalate into the endothelial monolayer, and penetrate the liver parenchyma, the adoption of mesenchymal features appears to be essential [26]. Indeed, we observed that treatment of amoeboid cells OMM 2.5 with Rac1-enriched exosomes (derived from the parental Mel 270 cells) led to enhanced cell migration and mesenchymal features.

Accordingly, Onken and colleagues have shown that both modes of migration are required by UM cells for transendothelial migration (TEM), and invasion of the surrounding tissues [26]. They demonstrated that TEM occurs via a stepwise process of ameboid blebbing and mesenchymal lamellipodial protrusions governed by RhoA and Rac1 signaling, respectively. This pathway is downstream of oncogenic Gαq/11, which controls motility and TEM in combination with BAP1-regulated pathways.

Notably, none of the proteins in Cluster 1 related to cytoskeletal organization and cell motility, were found to be overrepresented in EVs derived from the metastatic OMM 2.5 cell line. However, outside of these nodes but also ascribed to Cluster 1, we found proteins such as FN1 and vimentin that promote cell adhesion and motility, significantly increased in OMM 2.5-derived EVs [61].

It is well known that for migration to occur, a cell protrusion must form and then attach to its surroundings. Integrins, a major family of migration-promoting receptors, mediate adhesion to the ECM, link with actin filaments, and activate migration-related signaling molecules [44]. Consistently, a specific repertoire of integrins such as ITGα5, ITGα_V_, ITGα4, ITGα3, ITGβ3, and ITGβ1 was found upregulated in OMM 2.5 cells and their derived EVs (Figure 4) and assigned to Cluster 1 according to their interactions with other members. The enrichment of integrins in metastatic cell-derived exosomes was particularly interesting because it is known that exosomal surface integrins mediate the organ-specificity of metastasis, favoring circulating exosomes to fuse with target cells and ECM molecules in particular organs, settling and initiating the pre-metastatic niche formation [33]. The authors have shown the liver tropism of exosomes carrying α_V_β_5_, which preferentially binds to liver Kupffer cells. Thus, it is not surprising that we and others found this integrin significantly increased in exosomes derived from UM cell lines [16,19].

Notably, we did not find an exclusive enrichment of proteins related to cell cycle and proliferation in primary Mel 270-derived EVs, as reported by Tsering and colleagues [19]. Indeed, we did find overexpression of cell-cycle proteins in both EV-derived cell lines. For example, CDK2 and CDK5 (cyclin-dependent kinases), both belonging to Cluster 2 in the functional network [62,63], were found to be distinctly overrepresented in Mel 270 and OMM 2.5-derived EVs. In addition, OMM 2.5-derived exosomes were enriched in proteins involved in UM metastasis, such as GDF-15 [64], and transcription factors governing the neural crest and neural development (i.e., GAS7) [65]. Among others, we also found proteins such as MAGED2, a melanoma-associated antigen that has also been involved in cell cycle progression, modulation of DNA damage response, and melanoma metastasis [66]. There was also upregulation of Matrin-3, a downstream component of the FGF2 pathway that mediates melanoma cell proliferation and survival [67], and BUB3, a mitotic checkpoint regulator [68].

Remarkably, the overexpression of proteins associated with metastatic risk was not exclusive to EVs derived from the OMM 2.5 cell line. Indeed, we and others found some proteins to be increased, such as HSP90 and ENO1, across both the primary and metastatic cell lines [19], while several immunoregulatory proteins that may facilitate the evasion of immune attacks like CEACAM1 [69], CD276 [70], angiogenesis-related, and members of the VEGFA: VEGFR2 pathway were shown to be overrepresented in Mel 270-derived EVs.

Finally, although metabolism was not our focus, it is important to mention that EVs derived from OMM 2.5 contain a significant enrichment of proteins related to mitochondrial activity, pyruvate uptake, and ATP activity, as well as the ubiquitin-proteasome pathways and membrane trafficking (shown in Clusters 2 and 4).

### 4.2. Contribution of EVs Secreted by UM Cells to Pre-Metastatic Niche Formation and Liver Colonization

Increasing evidence strengthens the consensus that exosomes enact specific functions during tumor progression in narrow crosstalk with distinct microenvironments [8,71]. Accordingly, we aimed to explore the contribution of EVs secreted by UM cells to pre-metastatic niche formation and liver colonization for further metastasis development. Previous studies have demonstrated the hepatotropism of UM-derived exosomes upon injection into the retro-orbital sinus of immunodeficient mice [16,72]. Ambrosini and colleagues showed that the uptake of UM-derived exosomes by hepatocytes led to the release of pro-metastatic molecules such as MIF and CXCL1, an increase in fibronectin deposits, and the activation of MET, ERK, AKT, and STAT3 cell signaling pathways [16]. Furthermore, Piquet and colleagues have demonstrated that UM-derived EVs increase endothelial tube formation ability in vitro [73]. Seitz and colleagues attributed the pro-tumorogenic effect of HHSCs on UM cells to the aberrant fibroblastic growth factor (FGF) signaling elicited by FGF9 [15]. Not less important for the homing of UM to the liver is the induction of inflammatory mediators and pro-fibrogenic interleukins [74].

Of particular interest are the changes in the function of HHSCs mediated by tumor-derived exosomes that may enhance metastatic growth in the liver [37,72]. Our study also revealed the crucial contribution of liver macrophages to this process. The uptake of UM-derived exosomes by THP-1 macrophages, previously treated with PMA for 36 h, led to increased mRNA levels of surface markers such as *CD163*, indicative of polarization to an M2-like phenotype. The detection of M2 cytokines and bioactive molecules induced at the mRNA level by melanoma and pancreatic cancer exosomes revealed similar findings [36,75]. These molecules would be expected to promote a variety of pro-tumor functions such as growth, angiogenesis, invasion, immunosuppressive properties, and metastasis.

Importantly, we detected increased levels of *TGFβ* isoforms, *CTGF,* and *PDGFβ* transcripts in macrophages upon treatment with UM exosomes. Among these cytokines, TGFβ plays a master role in activating and transdifferentiating quiescent HHSCs into motile myofibroblasts [76,77]. In this study, we have co-cultured THP-1 macrophages with LX2 stellate cells in a non-contact-dependent manner and in the presence or absence of UM-derived exosomes. In agreement with previous reports on pancreatic cancer exosomes [23,78], we observed that UM-derived exosomes triggered the transactivation of HHSCs, which produced α-SMA and increased amounts of extracellular matrix (ECM) proteins such as FN1, FN-EDA, and *Col1A1* as compared with controls. Remarkably, these transcripts were not observed in single cultures of LX2 stellate cells or in the presence of a TGBβR inhibitor (Supplementary Figure S3). Thus, although the co-culture conditions do not mirror the organ environment-specific signals, this approach reveals the crosstalk between macrophages and stellate cells in the construction of a metastasis-supporting fibrotic microenvironment in the liver [7]. In turn, the fibrotic microenvironment may mediate the recruitment of bone marrow-derived macrophages and neutrophils, thus completing the formation of the pre-metastatic niche [23].

Finally, we assessed that HHSCs activated by co-cultures with macrophages in the presence of exosomes promote UM cell adhesion and migration, which may enhance metastatic growth in the liver. Current studies are deciphering the involvement of FAK-MMP9 signaling and the specific ECM components or growth factors secreted by activated HHSCs responsible for the activation of this signaling pathway.

Our results highlight the importance of identifying cargo proteins in UM-derived exosomes that contribute to tumor metastasis in vivo, acting on different steps of the process, and conveying changes in the UM migratory modes and transdifferentiation of HSCCs.

These findings provide new insights into the UM exosome’s contribution to liver metastasis and encourage further investigations on clinical samples to validate the results. Thus, UM-derived exosomes might be used as dual diagnostic and therapeutic tools. Therapeutic strategies conducted to abolish the uptake of UM exosomes or target their triggered signaling pathways might be helpful in hindering metastatic dissemination, whose consequences worsen the prognosis of these patients.

## 5. Conclusions

Our results indicate that the protein cargo of UM-derived exosomes impinges on distinct regulatory cellular functions on recipient cells (including both the tumor cells and the liver resident cells) that are crucial for metastatic success.

In this study, we performed a comparative proteomic analysis of exosomes isolated from UM Mel 270 cells and its isogenic OMM 2.5 metastatic cell line derived from the same patient. Using bioinformatic tools, we built a functional biological network using the 692 proteins whose representation in exosomes was statistically significant between the two groups, defining a cluster of proteins with specific functions related to cell detachment from the primary tumor (Rac1-enriched exosomes) or those that dictate liver specificity (ITGα_V_ and ITGβ5-enriched exosomes). Exosome treatments of UM cells revealed the plasticity of these cells, triggering a switch between mesenchymal and amoeboid modes of migration that facilitate the movement across distinct substrates and microenvironments.

Furthermore, we demonstrate the important role of liver macrophages in the transactivation of HHCSs by UM-derived exosomes in orchestrating the pro-fibrotic and pro-inflammatory microenvironment that enhances the adhesion and migration of UM cells, thus favoring liver colonization.

## Data Availability

Data will be available upon request.

## Supplementary Materials

The following supporting information can be downloaded at: https://www.mdpi.com/article/10.3390/cancers16172977/s1, File S1: The original Western blot figures; Figure S1: Acronyms (Id) of proteins belonging to Cluster 1 that are significantly modulated between Mel 270- and OMM 2.5- derived exosomes. Each protein received a consecutive number (1 to 221) and is plotted according its Log2Fold Change (7 to −7); Figure S2: Matrix Metalloproteinases (MMP) activities showed no significant differences between Mel 270 and OMM2.5 cells; Figure S3: Direct treatment of LX2 cells with uveal melanoma-derived exosomes failed to induce LX2 gene reprogramming; Table S1: Antibodies used; Table S2: Oligonucleotides used (qPCR); Table S3: EV PROTEINS DIFF MEL270 &OMM25; Table S4: EV prot common inMel270 &OMM2.5; Table S5: Figure 1J,K BP-Fold-Enrichment; Table S6: statistics. References [79,80] are cited in the Supplementary Materials.
