# Supplementary material for: New Insights into the Exosome-Induced Migration of Uveal Melanoma Cells and the Pre-Metastatic Niche Formation in the Liver
Source: Cancers (Basel). 2024 Aug 27;16(17):2977. doi: 10.3390/cancers16172977 (PMC11394004; doi:10.3390/cancers16172977)

RAW DATA FIG 1D

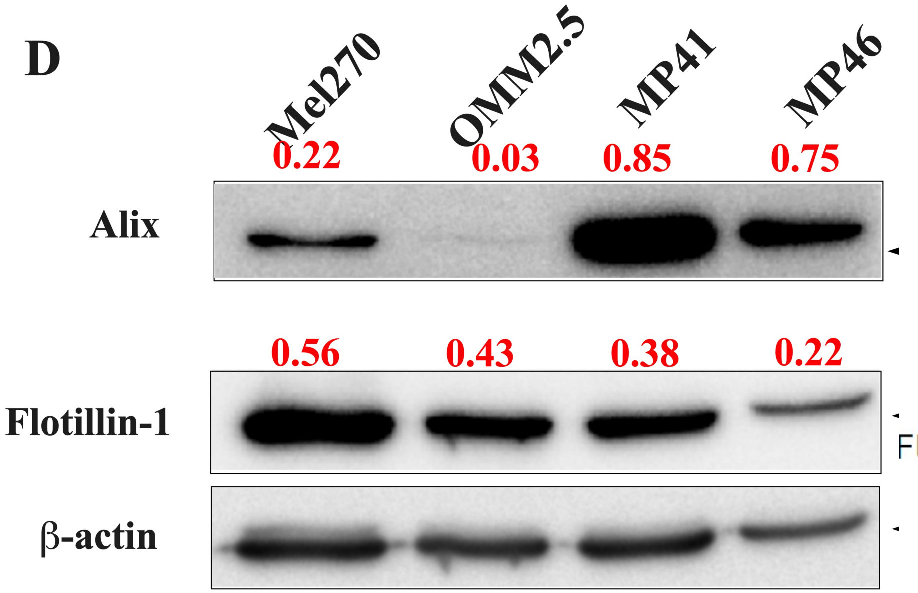

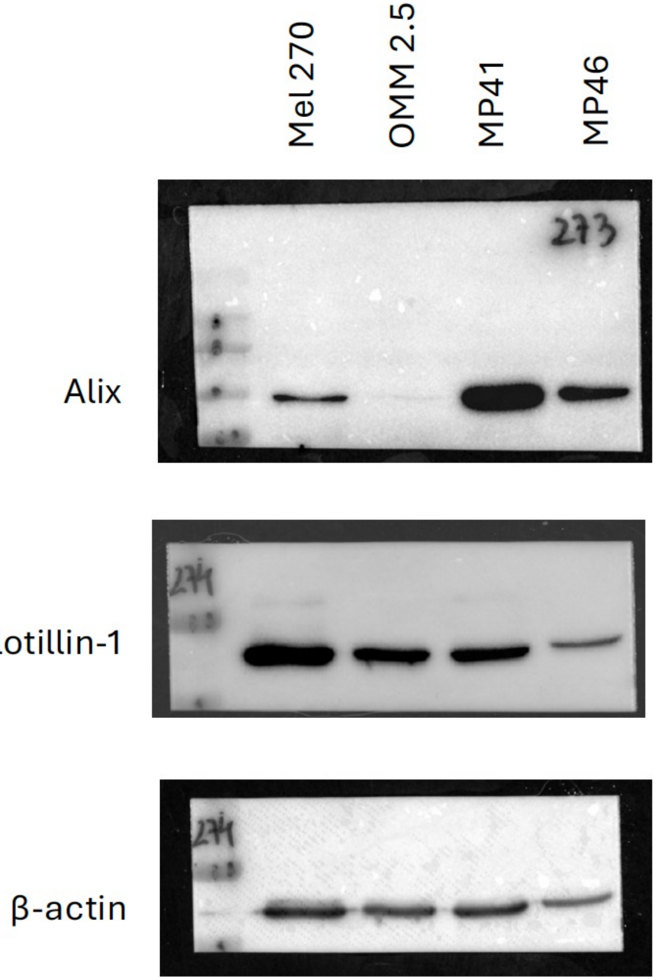

RAW DATA FIG 3G

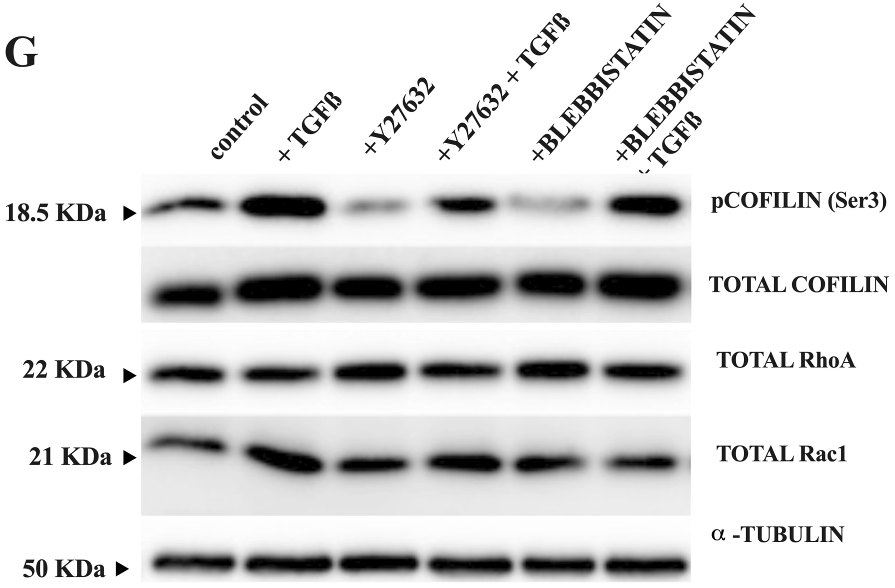

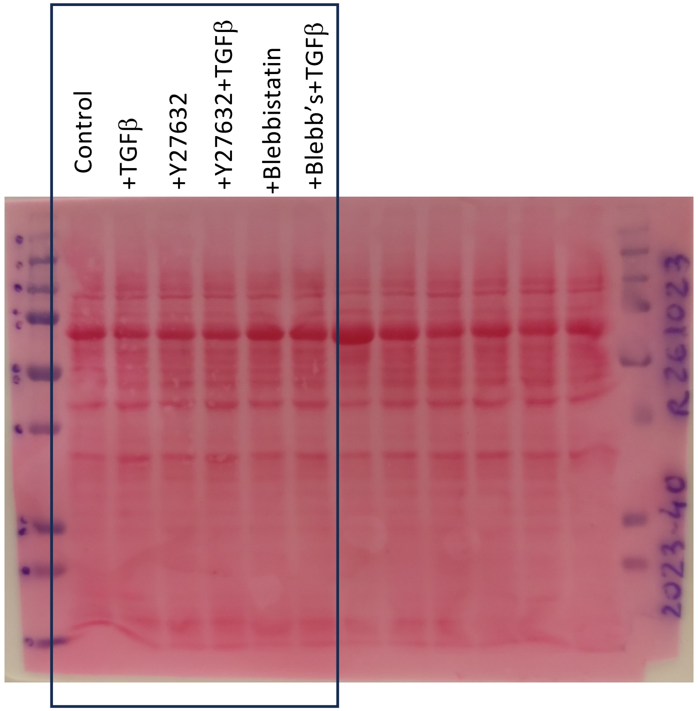

OMM 2.5 (2D CULTURE)

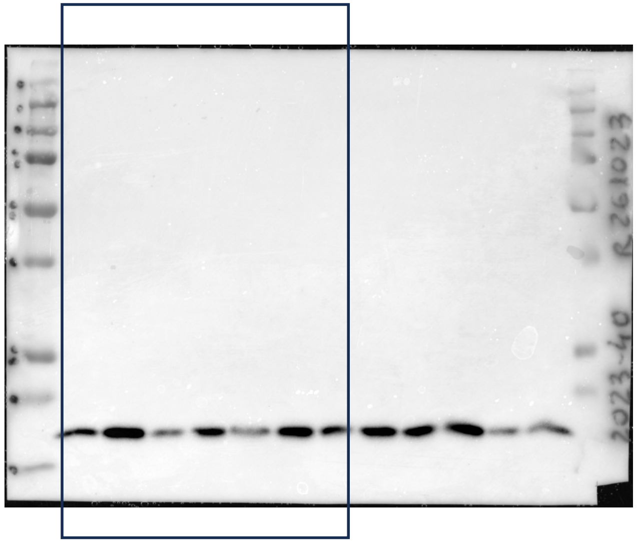

Phospho-Cofilin (Ser3)

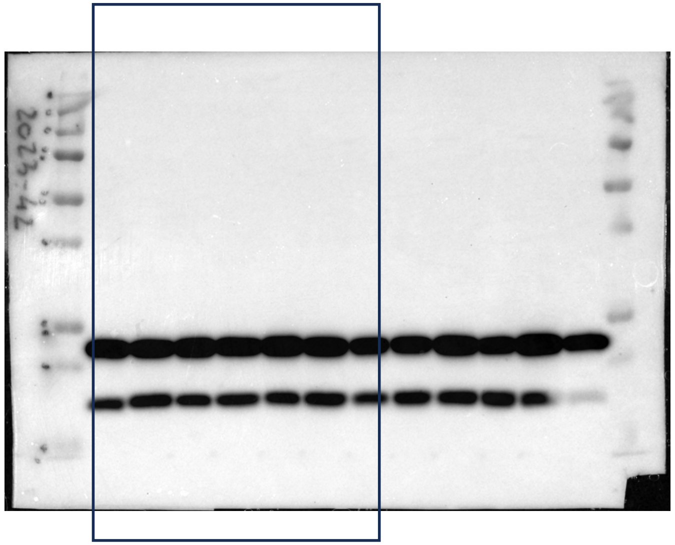

Cofilin Total

RAW DATA FIG 3G

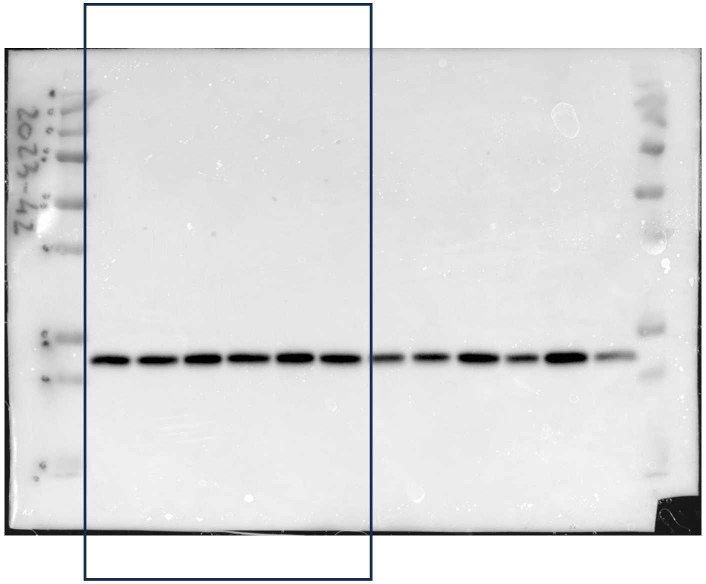

RhoA

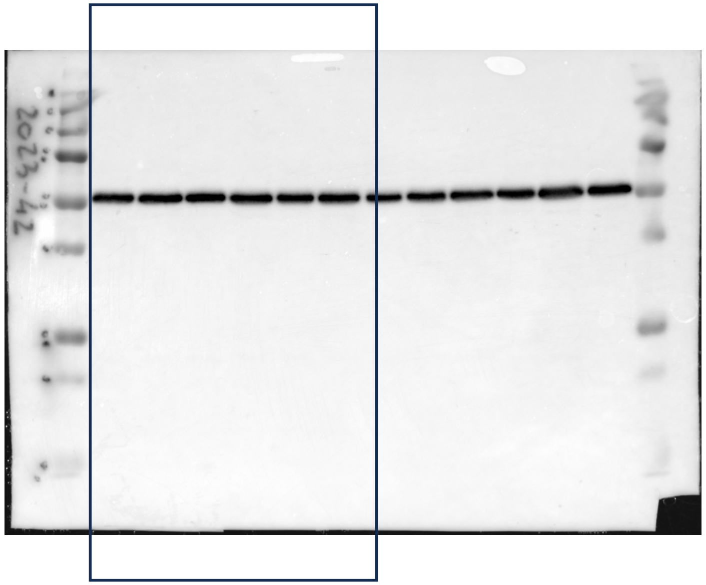

αTubulin

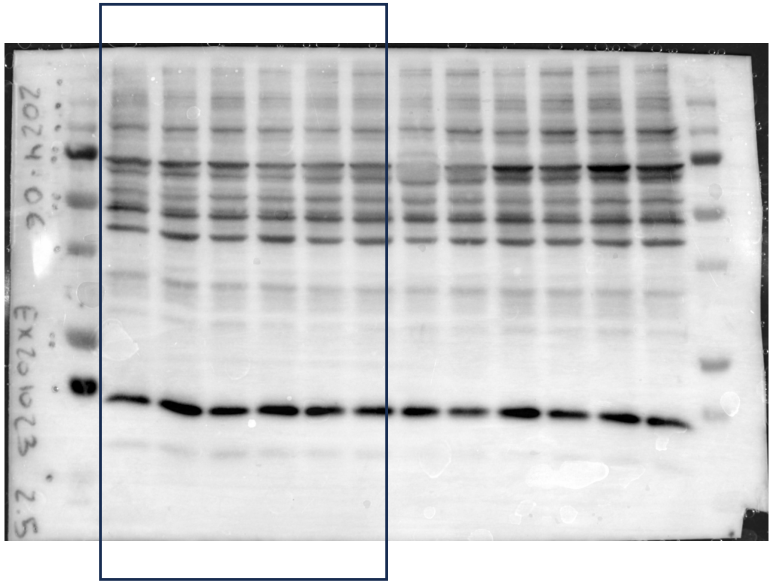

Rac1

RAW DATA FIG 6K

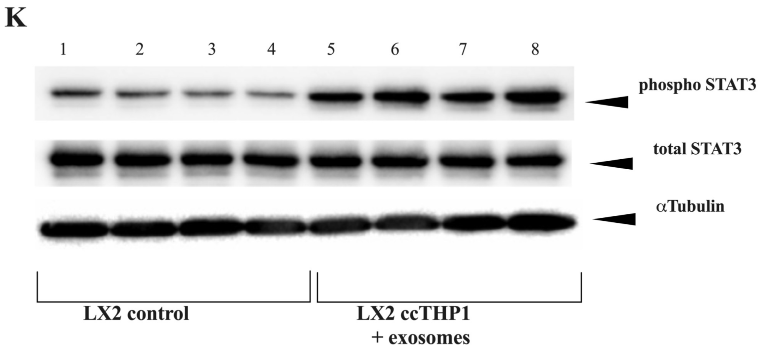

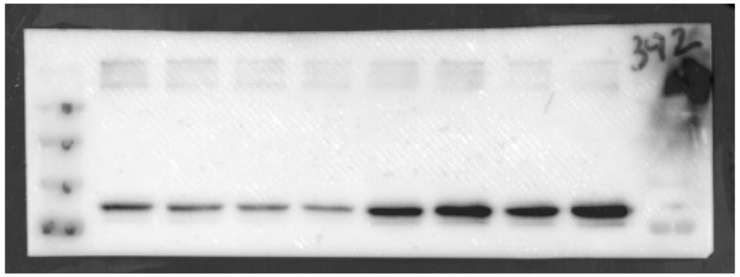

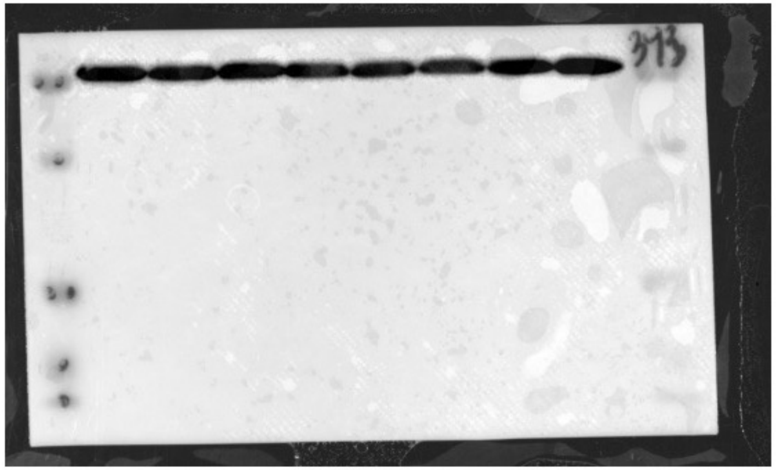

RAW DATA FIG 4B

B

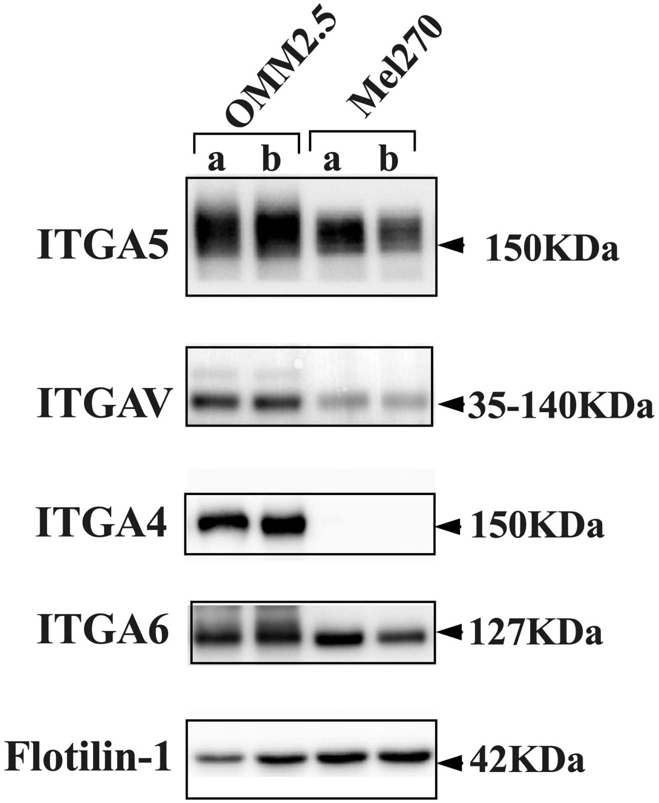

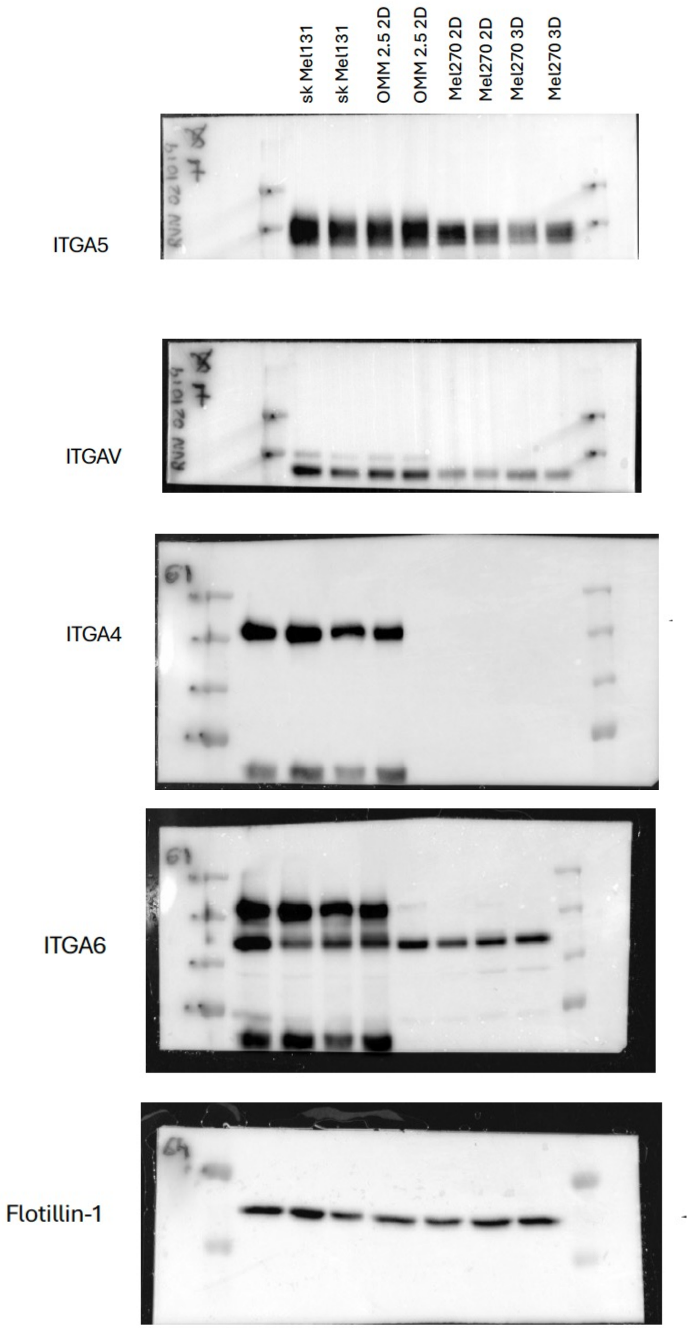

Supplement: Supplementary file 1 [file cancers-16-02977-s001.zip › cancers-3159344-File S1.pdf]
