# Supplementary material for: New Insights into the Exosome-Induced Migration of Uveal Melanoma Cells and the Pre-Metastatic Niche Formation in the Liver
Source: Cancers (Basel). 2024 Aug 27;16(17):2977. doi: 10.3390/cancers16172977 (PMC11394004; doi:10.3390/cancers16172977)
Supplement: Supplementary file 1 [file cancers-16-02977-s001.zip › Figures S1-S3.pdf]

PROTEINS IN CLUSTER #1

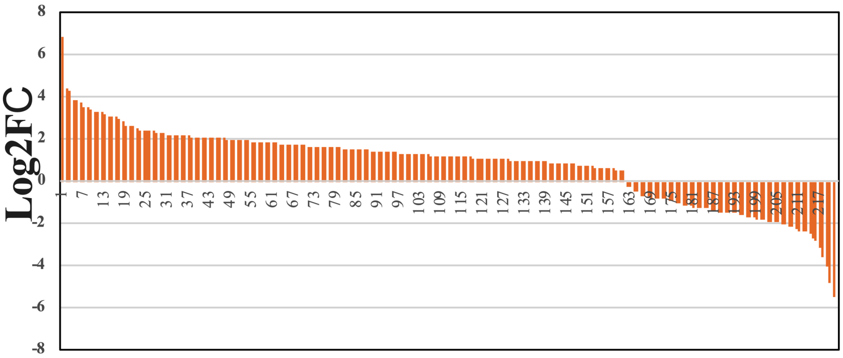

id 1-162: SH3PXD2B MMP14 PLXNA1 DBN1 APOE SNX9 MYH10 ANXA6 FLNB STX7 EPB41 STXBP1FN1 FAM49B TMED2HSPG2 SEC23A MFGE8 VAMP8 MYO1B ATP2B1 STX3 GNAI3 VAMP7 CORO1C TMED10 SDC4 BSG TAGLN2 CD47 LGALS3 NCKAP1 IST1 EEA1 SPTBN1 RALA LAMP1 IQGAP1 DOCK7 EPB41L3 VPS26A CAPZB GNA13 VPS35 TWF2 EPHA2 TFRC AHNAK PAG1 RAB7A GNA11 GNAS SLC16A1 CYFIP1 SPTAN1 TPM4 PDCD6 RAC1 RAC3RAC2 SCARB1 FARP1 VTA1 ARPC4 SNX2 RAB35 ARPC2 ACTR3 ERBIN GNG12 SLC3A2ARPC1B GNAQ ACTR2 CD63 DBNL MYO1C SEC22B STMN1 PTPRA TBC1D10A CLTC FTH1 YKT6 PIP4K2A NPC1 RAB21 L1CAM SPAG9 TJP1 CAPZA1 NAPG IGF1R VPS4B FSCN1 SNX12 RAB14 ARHGEF7 SNX6 EPS8 CTTN RAB5B RAB10 ACTN1 BRK1 TWF1 GDI2 ANXA4 GYPC ARPC5 RAB1A ABI1 RHOG ARHGAP17 PDCD6IP PLS3 NPTN STX4 STXBP3 RAB1B GNB1 AP2M1 RAB2A S100A11 MAPRE1 GNAI2 STX8 CDC42 CORO1B MPP1 WASF2 APEH SORD NAPA VAMP3 RALB VCL SEC16A ANXA7 EPS15 PAK2 S100A10 CHMP3 GDI1 GRB2 FLNA ANXA2 CAP1 CALD1 CHMP2A ARHGDIA ANXA11 PFN1CAPZA2 RAB5C TLN1 CD44 ARF4 DNM2 VPS4A VPS29

id 163-221: TGOLN2 MYH9 RAB11B RAB11A PXN ITGAV DCTN1 KIF5B CLTA SH3GL1 ARCN1 DCTN2 SEPTIN8 ARHGAP1 COPB2 ICAM1 EXOC7 ANXA1 ITGB1 MYL9 MYL12A MYL12B FASN ITGB5 ITGA3 VPS45 RAB11FIP5 ADGRG1 IGF2R PREP GLB1 CTSDSEPTIN2 DYNC1LI2 RAB6A COPE SNX3 ATIC CSK BUB3 CD99 TPP1 SEPTIN10 MAGED2 S100A13 DYNC1LI1 KLC1 NRP2 GBA MYOF LGALS1 SQSTM1 GPNMB S100A6 CTSB ACSL4 SERPINE2 TIMP3 NT5E

Supplementary Figure S1:
Acronyms (Id) of proteins belonging to Cluster #1that are significantly modulated between Mel 270- and OMM 2.5- derived exosomes. Each protein received a consecutive number (1 to 221) and is plotted according its Log2Fold Change (7 to -7).

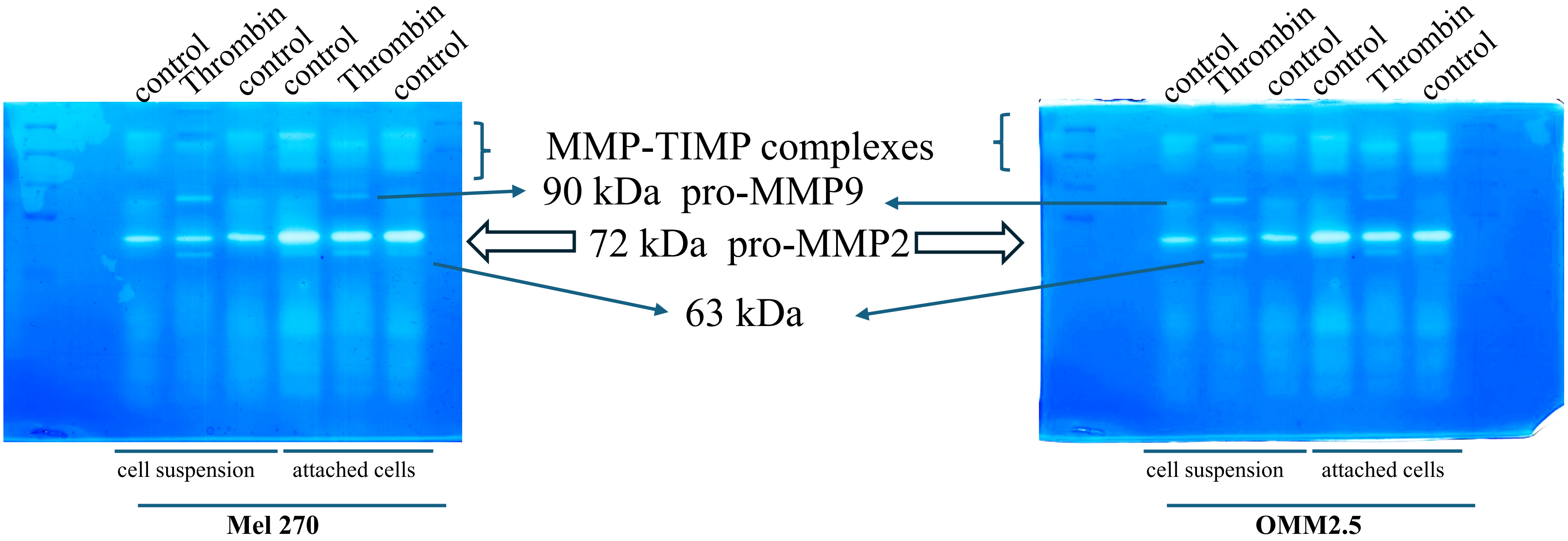

Supplementary Figure S2

Matrix Metalloproteinases (MMP) activities showed no significant differences between Mel 270 and OMM2.5 cells. Gelatin zymography of conditioned media from Mel 271 (*left* panel) and OMM 2.5 (*right* panel) cultured in single cell suspension or attached to plastic. Here are shown secreted gelatinases from controls (untreated cells), and Thrombin-treated cells for 30 h. Thrombin induced MMP-9 expression and the cleaved 63 kDa catalytic form generated by MT1-MMP (MMP-14) (1,2). Cleared bands show the position of secreted gelatinases on dark SDS-gelatin gel stained with Coomassie blue. The images are representative of two independent experiments.

(1) Chang C.J. et al., Biochem. and Biophysical Res Comm.(2009) vol. 385 (2), 241.
(2) Lafleur et al., Biochem. J. (2001) 357, 107.

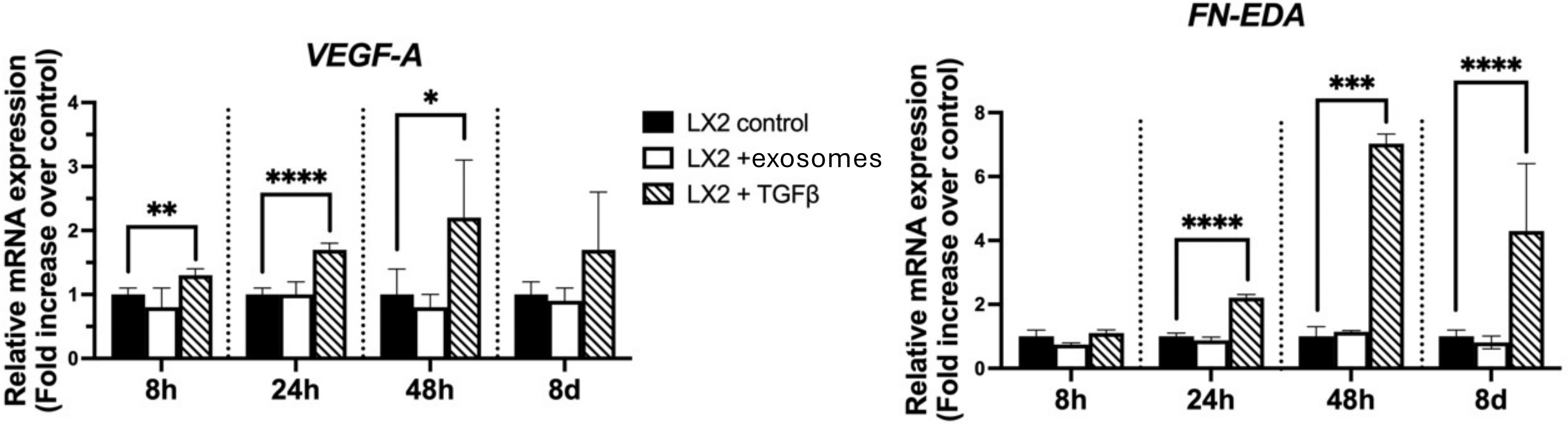

Supplementary Figure S3

Direct treatment of LX2 cells with uveal melanoma-derived exosomes failed to induce LX2 gene reprogramming.

Boxplots showing the mRNA expression of VEGF-A, and FN-EDA in LX2 cells after the indicated experimental conditions. Cells were collected after 72 hours of treatment. Gene expression is presented relative to the RPL32 gene. Data are mean ± SD, *p<0.05, **p<0.01, ***p<0.001, ****p<0.0001, ns, not significant by One-way ANOVA.
