# Supplementary material for: New Insights into the Exosome-Induced Migration of Uveal Melanoma Cells and the Pre-Metastatic Niche Formation in the Liver
Source: Cancers (Basel). 2024 Aug 27;16(17):2977. doi: 10.3390/cancers16172977 (PMC11394004; doi:10.3390/cancers16172977)
Supplement: Supplementary file 1 [file cancers-16-02977-s001.zip › Tables S1 and S2.pdf]

Supplementary Materials:

Supplementary Table S1: Antibodies used.

PRIMARY ANTIBODIES	SOURCE	REFERENCE	Dilution (WB/IHQ)
ß-Actin-HRP	Abcam	ab49900	1:1000
Flotilin-1	BD Transduct. Lab	610821	1:1000
Alix	Cell Signaling	2171	1:1000
CD9	Abcam	ab92726	1:2000
p-Cofilin	Abcam	ab12866	1:1000
Cofilin	Abcam	ab134963	1:1000
FN-EDA	Abcam	ab6308	1:200
RhoA	Cytoskeleton Inc.	ARH05	1:1000
Rac-1	Cytoskeleton Inc.	ARC03	1:250
ITG$\alpha_v\beta_5$	BIOSS antibodies	bs-1356R	1:200
ITG$\alpha_v\beta_3$	BIOSS antibodies	bs-1310R	1:200
ITGA4	Abcam	Ab81280	1:1500
ITGA6	Novus Biological	NBP1-85747	1:250
Phospho-MLC2 (thr18/Ser19)	Cell Signaling	3674	1:1000 (IF)
p44/42 MAP Kinase	Cell Signaling	9102	1:1000 (WB)
AKT	Cell Signaling	9272	1:1000
α-Tubulin	Sigma	T6074	1:10000

SECONDARY ANTIBODIES	SOURCE	REFERENCE	Dilution
anti-mouse HRP	Dako	P0260	1:10000
anti-rabbit HRP	Dako	P0448	1:10000
anti-rabbit Alexa Fluor 568	Jackson	711-165-152	1:200
Anti-mouse Alexa Fluor 647	Jackson	715-605-151	1:200

Supplementary Table S2: Oligonucleotides used (qPCR).

RP L32	**Forward**	**GAT CTT GAT GCC CAA CAT TGG TTA TG**
	Reverse	GCA CTT CCA GCT CCT TGA CG
CD63	Forward	AGC AGA TGG AGA ATT ACC C
	Reverse	CTC CCA ATC TGT GTA GTT AG
RAB27A	Forward	GGCATTGATTTCAGGGAAAA
	Reverse	CGCTGTCGTTAAGCTACGAA
IL8	Forward	CTCTTGGCAGCCTTCCTGATT
	Reverse	TATGCACTGACATCTAAGTTCTTTAGCA
TGFß1	Forward	GCACGTGGAGCTGTACCAGAAA
	Reverse	CGCACAACTCCGGTGACATCAAAA
TGFß2	Forward	TACTACGCCAAGGAGGTTTACAAA
	Reverse	TTGTTCAGGCACTCTGGCTTT
TGFß3	Forward	GTCACACCTTTCAGCCCAAT
	Reverse	GCAGTTCTCCTCCAAAGTTGC
FN1	Forward	CAATGTGGGTCCCTCTGTCT
	Reverse	CAGGCTGCAGTGTGGTAAAG
FN-EDA	Forward	GGAGAGAGTCAGCCTCTGGTTCAG
	Reverse	TCTGCAGTGTCTTCTTCACC
a SMA	Forward	AGAGTTACGAGTTGCCTGATG
	Reverse	GCTGTTGTAGGTGGTTTCATG
CYCLIN D	Forward	TTTGTCCAGCCCACAGTGTT
	Reverse	ACGCCATAGCAATTCACCCA
COL1A1	Forward	ATGGATTCCAGTTCGAGTATGGC
	Reverse	CATCGACAGTGACGCTGTAGG

AURKA	Forward	CCAGGGACCTCATTTCAAG **A**
	Reverse	TTGTTTTGGCAATTTGATGG
CYCLIND1	Forward	CACTTGCATGTTCGTGGCCTCT
	Reverse	CTCCTCCGCCTCTGGCATTTT
IL6	Forward	ATGCAATAACCACCCCTGAC
	Reverse	ATCTGAGGTGCCCATGCTAC
VEGF	Forward	GCATTGGAGCCTTGCCTTG
	Reverse	CCTTCGTGATGATTCTGCCCT
CTGF	Forward	CGTTCAAGCATGAAATGGA
	Reverse	GAAACAAATGCTTCCAGGTGA
MIF	Forward	CACAGTGGTGTCCGAGAAGTCA
	Reverse	TCCCTGCTGCTATTAGGCGAA
IL1ß	Forward	GCTGGAGAGTGTAGATCCCAAA
	Reverse	GTTATATCCTGGCCGCCGCCTTT
MMP9	Forward	GCGGAGATTGGGAACCAGCTG TA
	Reverse	GACGCGCCTGTGTACACCCACA
PDGFD	Forward	GTGGAGGAAATTGTGGCTGT
	Reverse	CGTTCATGGTGATCCAACTG
PRRX1	Forward	CTGATGCTTTTGTGCGAGAA
	Reverse	ACTTGGCTCTTCGGTTCTGA
ZEB1	Forward	AAGAATTCACAGTGGAGAGAAGCCA
	Reverse	CGTTTCTTGCAGTTTGGGCATT
CD44	Forward	AAGGTGGAGCAAACACAACC
	Reverse	ACTGCAATGCAAACTGCAAG
IL10	Forward	TGCCTTCAGCAGAGTGAAGA
	Reverse	GCAACCCAGGTAACCCTTAAA
CCL2	Forward	ACAAGCAAACCCAAACTCCG
	Reverse	AAACAGGGTGTCTGGGGAAA
CCL20	Forward	CTGTGACATCAATGCTATCATCTTTCA
	Reverse	CTTGGGCTATGTCCAATTCCATTCC
CD163	Forward	GAGTCCCTTCACCATTACTGTG
	Reverse	GACTTTCACTTCCACTCTCCC
CXCL12	Forward	GAAAGCCATGTTGCCAGAG
	Reverse	TGAATCCACTTTAGCTTCGGG
